# stelfi: An R package for fitting Hawkes and log‐Gaussian Cox point process models

**DOI:** 10.1002/ece3.11005

**Published:** 2024-02-13

**Authors:** Charlotte M. Jones‐Todd, Alec B. M. van Helsdingen

**Affiliations:** ^1^ Department of Statistics University of Auckland Auckland New Zealand

**Keywords:** Hawkes process, log‐Gaussian Cox process, point‐referenced data, self‐exciting, spatiotemporal, Template Model Builder

## Abstract

Modelling spatial and temporal patterns in ecology is imperative to understand the complex processes inherent in ecological phenomena. Log‐Gaussian Cox processes are a popular choice among ecologists to describe the spatiotemporal distribution of point‐referenced data. In addition, point pattern models where events instigate others nearby (i.e., self‐exciting behaviour) are becoming increasingly popular to infer the contagious nature of events (e.g., animal sightings). While there are existing R packages that facilitate fitting spatiotemporal point processes and, separately, self‐exciting models, none incorporate both. We present an R package, stelfi, that fits spatiotemporal self‐exciting and log‐Gaussian Cox process models using Template Model Builder through a range of custom‐written C++ templates. We illustrate the use of stelfi's functions fitting models to Sasquatch (bigfoot) sightings data within the USA. The structure of these data is typical of many seen in ecology studies. We show, from a temporal Hawkes process to a spatiotemporal self‐exciting model, how the models offered by the package enable additional insights into the temporal and spatial progression of point pattern data. We present extensions to these well‐known models that include spatiotemporal self‐excitation and joint likelihood models, which are better suited to capture the complex mechanisms inherent in many ecological data. The package stelfi offers user‐friendly functionality, is open source, and is available from CRAN. It offers the implementation of complex spatiotemporal point process models in R for applications even beyond the field of ecology.

## Graphical abstract

We introduce the R package stelfi, available from the Comprehensive R Archive Network. This package allows users to fit temporal self‐exciting Hawkes models, spatial and spatiotemporal log‐Gaussian Cox process models and self‐exciting spatiotemporal models. The functionality of stelfi is illustrated using Sasquatch (bigfoot) sightings data shipped with the package.

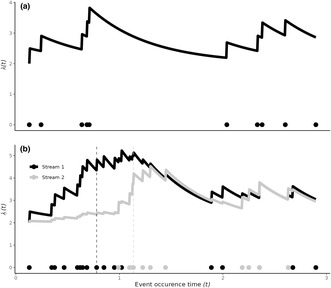

## 1 INTRODUCTION

Point patterns are data that describe the locations of objects or events in space and time. For example, the location of a particular species, its nest or the timestamp of its sighting are all point pattern data. A point pattern is a random variable, a single realisation of some assumed point process that describes the characteristics of the observed spatiotemporal distribution (Baddeley et al., 2015; Diggle, 2013; Illian et al., 2008; Ripley, 1981).

Point pattern data are widely seen in ecology (Ben‐Said, 2021; Ripley, 1987; Velázquez et al., 2016; Wiegand & Moloney, 2014). With rich spatial and temporal data becoming increasingly available ecologists require progressively sophisticated point process models to capture the structure and dependencies inherent in such data. Summary statistics (e.g., Ripley's K‐function) are only able to give ecologists a synoptic description of the data (e.g., the degree of deviation from a homogeneous Poisson process). In reality, an ecologist's interest lies in inferring the effect of environmental covariates and/or characteristics of the points (i.e., marks) on the spatiotemporal distribution of the ecological objects (Velázquez et al., 2016; Wiegand & Moloney, 2014).

Modelling spatial point pattern data as a log‐Gaussian Cox process (Cox & Isham, 1980; Møller et al., 1998) is a popular choice for many ecological applications (Illian et al., 2012; Serra et al., 2014; Simpson et al., 2016; Soriano‐Redondo et al., 2019). However, in many situations, the occurrence times of events are self‐exciting (i.e., the occurrence of an event instigates another). One well‐known model describing this phenomenon is a Hawkes process (Hawkes, 1971a, 1971b); these models, and their extensions, are often seen in seismology (Ogata, 1988), criminology (Park et al., 2021; Zhuang & Mateu, 2019) and finance (Bacry et al., 2015; Hawkes, 2018). Recently, however, they have begun to see some use in ecology (Gupta et al., 2018; Nakagawa et al., 2019).

Additionally, the characteristics (i.e., marks) of the points may depend on the locations of the objects. For example, certain species of shrub may be more likely to cluster together (higher point intensity). This can be formulated similarly to a preferential sampling model where the log‐Gaussian Cox model assumed for the point pattern (the locations) is modelled jointly alongside the response (i.e., mark) (Diggle & Su, 2010).

We introduce the R package stelfi (Jones‐Todd & van Helsdingen, 2023), available from the Comprehensive R Archive Network (CRAN), which fits spatiotemporal point process models using Template Model Builder (Kristensen et al., 2016). Specifically, stelfi allows users to fit temporal self‐exciting Hawkes models (Hawkes, 1971a, 1971b), spatial and spatiotemporal log‐Gaussian Cox process models (Cox & Isham, 1980) and self‐exciting spatiotemporal models.

The most widely known and user‐friendly R packages that fit log‐Gaussian Cox processes include spatstat (Baddeley et al., 2015), lgcp (Taylor et al., 2013), and inlabru (Bachl et al., 2019) a wrapper for INLA (Lindgren & Rue, 2015). The package spatstat offers a comprehensive suite of methods to summarise and model point pattern data. When fitting these models, the user can choose either a minimum contrast, second‐order composite likelihood or a Palm likelihood approach. The packages lgcp and inlabru fit models using a Bayesian framework via Markov‐chain Monte Carlo (Robert et al., 1999) and Laplace approximation, respectively.

Just over a decade ago Rue et al. (2009) developed the Integrated Nested Laplace Approximation approach that uses Laplace approximation techniques to fit latent Gaussian models. A few years later, the Stochastic Partial Differential Equation approach was established by Lindgren et al. (2011). This methodology approximates the Gaussian field by a Markovian equivalent, a Gaussian Markov Random Field. Although INLA (Lindgren & Rue, 2015) does so, it is not necessary to use this Bayesian approach if you wish to make use of this framework. We use Template Model Builder (Kristensen et al., 2016); here, again, the Laplace approximation is used to approximate the integration across the random effects (Skaug & Fournier, 2006).

There are a number of R packages that fit temporal Hawkes process models: emhawkes (Lee, 2021) where estimation is based on the maximum likelihood method introduced by Ozaki (1979); hawkesbow (Cheysson, 2021), which fits a Hawkes process to discrete data by minimising the Whittle contrast; hawkes (Zaatour, 2014) that only allows users to evaluate the Hawkes likelihood for their own optimisation technique bayesianETAS (Ross, 2017), and most recently ETAS.inlabru (Naylor & Serafini, 2023; Serafini et al., 2023), allow users to fit epidemic‐type aftershock sequence models (a special case of a Hawkes process typically used to model the evolution of seismicity over time and space; Ogata, 1988) using Bayesian estimation techniques.

In contrast to the packages mentioned above, the R package stelfi uses custom Template Model Builder (Kristensen et al., 2016) templates written in C++ to fit spatiotemporal Hawkes and log‐Gaussian Cox models. Where appropriate, the Laplace approximation is used to approximate the integration over the random effects (Skaug & Fournier, 2006). We present extensions to the well‐known Hawkes and log‐Gaussian Cox process, including spatiotemporal self‐excitation and joint likelihood models, which are better suited to capture the complex mechanisms inherent in many ecological data.

The package stelfi is available on CRAN, with the development version on GitHub https://github.com/cmjt/stelfi. The novelty of the package is twofold (1) custom‐written C++ templates take advantage of the R package TMB (Kristensen et al., 2016), fitting models via maximum likelihood; (2) it facilitates the fitting of temporal and spatiotemporal self‐exciting point process models.

In the sections below we outline the range of spatial, temporal, and spatiotemporal point process models offered by stelfi and move on to illustrate their use by modelling Sasquatch sighting data, collected by the Bigfoot Field Researchers Organization (BFRO) (BFRO, 1995) and collated by Renner (2021). These data contain the temporal and spatial locations of claimed sightings of Sasquatch across the contiguous USA ($A∼7,771,155{\mathrm{km}}^{2}$) from 2000 to 2006. Although the data pertain to sightings of the likely fictional creature, colloquially known as Bigfoot, they are legitimate and share a structure common to many ecological examples (i.e., spatial and temporal coordinates). These data are shipped with the package stelfi as the object sasquatch.

## 2 METHODS AND FEATURES

The core functions of stelfi allow users to fit (1) temporal Hawkes point processes, with the extension of a user‐defined temporally varying background function and multiple correlated streams; (2) spatial or spatiotemporal log‐Gaussian Cox processes, with the extension of jointly modelling relevant marks; (3) self‐exciting spatiotemporal models where the spatial self‐excitement may be either time‐dependent or not.

Before we outline the functionality offered by stelfi we will first list the acronyms we will use, for brevity, from here on. Where necessary we also give a brief description of the context we assume, please see the references for the full definitions. All acronyms are commonly used in their respective literature.

- GRF, Gaussian Random Field, a collection of (typically 2D) Gaussian distributed random variables.
- GMRF, Gaussian Markov Random Field, the Markovian equivalent of a GRF (i.e., where the Markov property of conditional independence holds).
- LGCP, Log‐Gaussian Cox Process, a commonly used statistical model for clustered point pattern data, which includes a latent GRF (Cox & Isham, 1980).
- INLA, Integrated Nested Laplace Approximation, a Bayesian method for estimating parameters of latent Gaussian model (e.g., the LGCP) (Lindgren & Rue, 2015).
- SPDE, Stochastic Partial Differential Equation, an equation whose solution under certain conditions is the GMRF approximation to the assumed GRF (Lindgren et al., 2011).
- TMB, Template Model Builder, an R package for fitting statistical latent variable models (Kristensen et al., 2016).

### 2.1 The Hawkes processes

Hawkes processes (Hawkes, 1971a, 1971b) are self‐exciting temporal processes, where the occurrence of one event increases the probability of events in the near future. This makes them a particularly useful tool in describing clustering and interactions between events (i.e., where on event might induce the occurrence of another in near proximity). A Hawkes process has event (e.g., sighting of a species) times $0<\tau_{1}<\tau_{2}<\ldots <T$ where the first event occurs at $\tau_{1}$. After each event the intensity immediately increases, meaning that the occurrence of one event ‘excites’ another by some degree. Many ecological or environmental data are the result of some contagious effect. For example, the (random) sighting of a species might induce a sighting in the near future only because one is preinclinded to sight it. Modelling these data with a self‐exciting process enables the degree of contagion (i.e., self‐excitement) to be inferred.

**FIGURE 1:**
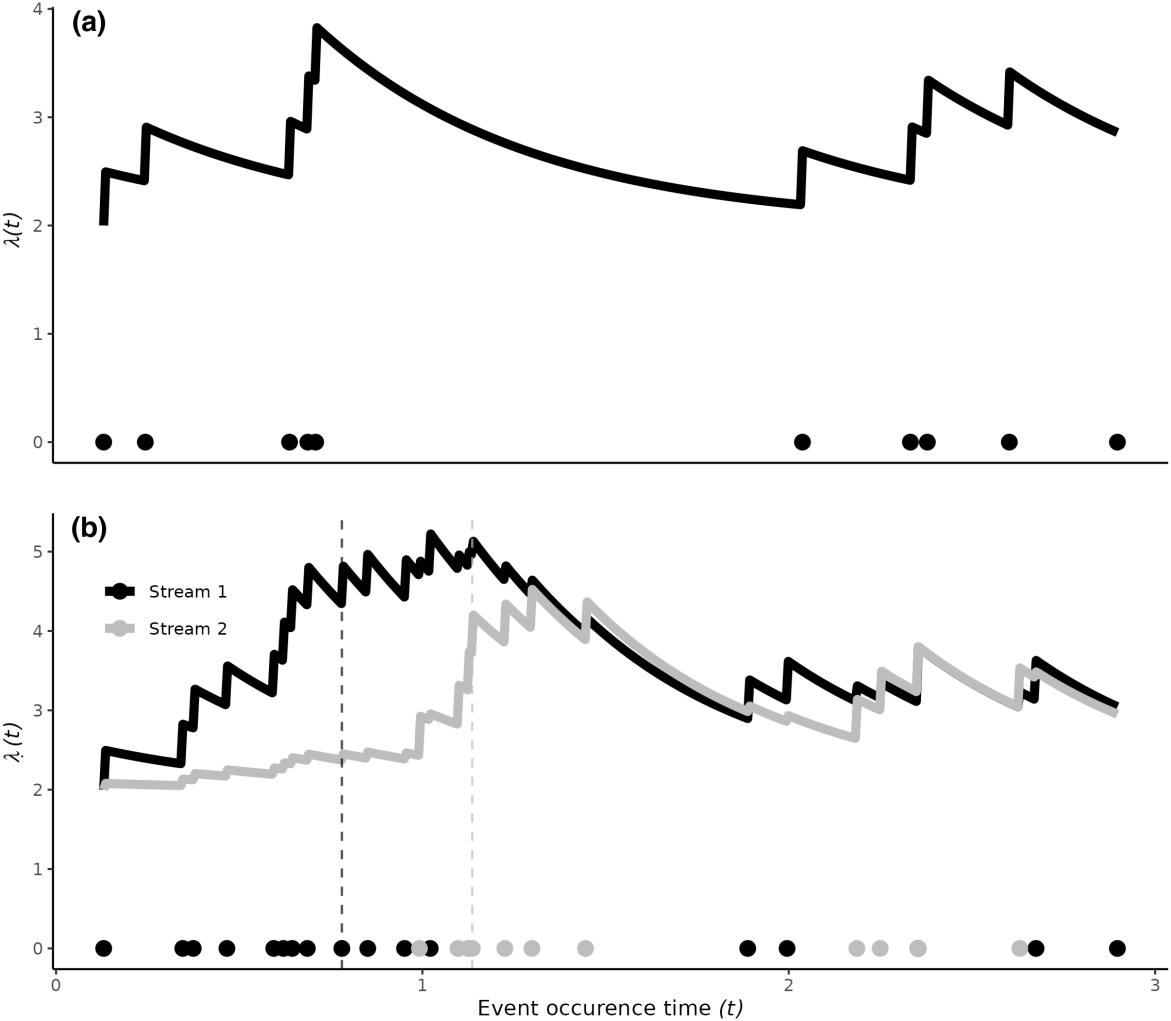
Plot (a) Realisation of a Hawkes process where the observed event occurrences are shown by the points. The Hawkes intensity, $\lambda \left(t\right)$, is given by the solid line, increases immediately after an event occurs and decays exponentially over time if no event is observed for some period. Plot (b) A realisation of a bivariate Hawkes process where the observed event occurrences from two streams are shown by the coloured points. The Hawkes intensities, $\lambda_{\text{Stream}1}\left(t\right)$ and $\lambda_{\text{Stream}2}\left(t\right)$, are given by the solid lines. Each intensity exhibits within‐ and between‐stream excitement. The black dashed vertical line shows that a Stream 1 event causes a jump in $\lambda_{\text{Stream}1}\left(t\right)$ as well as, to a smaller degree, $\lambda_{\text{Stream}2}\left(t\right)$; The grey‐dashed vertical line shows that a Stream 2 event again causes a jump both intensities. This bivariate Hawkes was simulated with greater within‐stream excitement (self‐excitement) than between‐stream excitement (cross‐excitement).

Figure 1 plot (a) shows a point pattern where the observed event occurrences are shown by the points along the *x*‐axis. The Hawkes intensity is given by the solid line and can be thought of as akin to the chance of observing an event at any time t, which increases immediately after an event occurs and decays exponentially over time if no event is observed for some period.

For current time t the conditional intensity function, $\lambda \left(t\right)$ in Figure 1 plot (a), is given by

$$\lambda \left(t\right)=\mu +\alpha \sum_{i:\tau_{i}<t}\exp\left(-\beta^{*}\left(t-\tau_{i}\right)\right).$$

Here μ is the background Poisson rate of the process. The term $\alpha \;\sum_{i:\tau_{i}<t}\exp\left(-\beta \;\left(t-\tau_{i}\right)\right)$ describes the historic temporal dependence (i.e., for times $\tau_{i}<t$, i=1,…,T). The parameter α is the increase in intensity immediately after the occurrence of an event, and β>0 controls the exponential decay of the intensity if no event has occurred. An extension of this includes mark information and is given by

(1) $$\lambda \left(t;m\left(t\right)\right)=\mu +\alpha \sum_{i:\tau_{i}<t}m\left(\tau_{i}\right)\exp\left(-\beta \left(t-\tau_{i}\right)\right).$$

Here $m\left(t\right)$ is the temporal mark that multiples the self‐exciting component of $\lambda \left(t;m\left(t\right)\right)$. The package stelfi implements additional extensions where (1) the background rate can vary in time where μ in Equation (1) becomes $\mu \left(t\right)$ according to some user‐defined function and (2) the process can be self‐inhibitive or self‐correcting (i.e., α<0). Examples of these extensions are given in the package's online gitbook https://cmjt.github.io/stelfi/.

### 2.2 A multivariate Hawkes process

A multivariate Hawkes process has multiple types of events (streams or threads) where the event occurrence of any type of event is influenced by all past events (i.e., event of any type). Consider the sightings example given in the section above, but now we have two sympatric (co‐occurring) species where after the (random) sighting of any one species the chance of observing either species immediately increases. This is an example of a bivariate Hawkes process where both self‐excitement (i.e., the chance of sighting a species increases immediately after observing it) and cross‐excitement (i.e., the chance of sighting a species increases immediately after observing a sympatric species) may exist. Fitting a multivariate Hawkes process enables the degree of the excitement within (self‐excitement) and between (cross‐excitement) event types to be inferred.

Figure 1 plot (b) shows a bivariate Hawkes process. The intensity of any stream, $\lambda_{\cdot }\left(t\right)$, increases immediately after any event occurs and decays exponentially over time if no event is observed for some period. The influence of one stream on another may differ. For example, in Figure 1 plot (b) the point pattern is simulated from a multivariate Hawkes process where the background rate, the within‐stream excitement, and the exponential decay for each are the same, however, the within‐stream influence is greater than the between‐stream influence. In plot (b) in Figure 1 this cross‐excitation effect is evident; for example, at the dashed horizontal lines, both intensities jump irrespective of the observed stream event.

Formally, the conditional intensity for the jth (j=1,…,N) stream is given by

(2) $$\lambda {\left(t\right)}^{j*}=\mu_{j}+\sum_{k=1}^{N}\sum_{i:\tau_{i}<t}\alpha_{\mathrm{jk}}e^{\left(-\beta_{j}*\left(t-\tau_{i}\right)\right)},$$

where $j,k\in \left(1,\ldots ,N\right)$. Here, $\alpha_{\mathrm{jk}}$ is the excitement caused by the kth stream on the jth. Therefore, α is an N×N matrix where the diagonals represent the within‐stream excitement, and the off‐diagonals represent the excitement between streams.

### 2.3 The log‐Gaussian Cox process

A LGCP (Møller et al., 1998) is a commonly used model for spatially clustered point pattern data. It is often called a hierarchical, or doubly stochastic, process as it is an inhomogeneous Poisson process (describing the number of points) with a (log) Gaussian random intensity process (describing the spatial dependence among points). The spatial distribution of points is conditionally independent given the Gaussian random intensity process, otherwise called a GRF.

Spatially clustered point pattern data occur often in ecological and environmental data. These clusters can be a result of many reasons (e.g., habitat preference, environmental covariates, over‐sampling), which may be known and measurable or not. Often, an ecologist's interest lies in inferring the effect of environmental covariates while accounting (somehow) for the remaining unexplained spatial structure. Fitting a LGCP enables the effect of covariates to be inferred while the GRF accounts for any unexplained residual spatial dependence (e.g., clustering).

**FIGURE 2:**
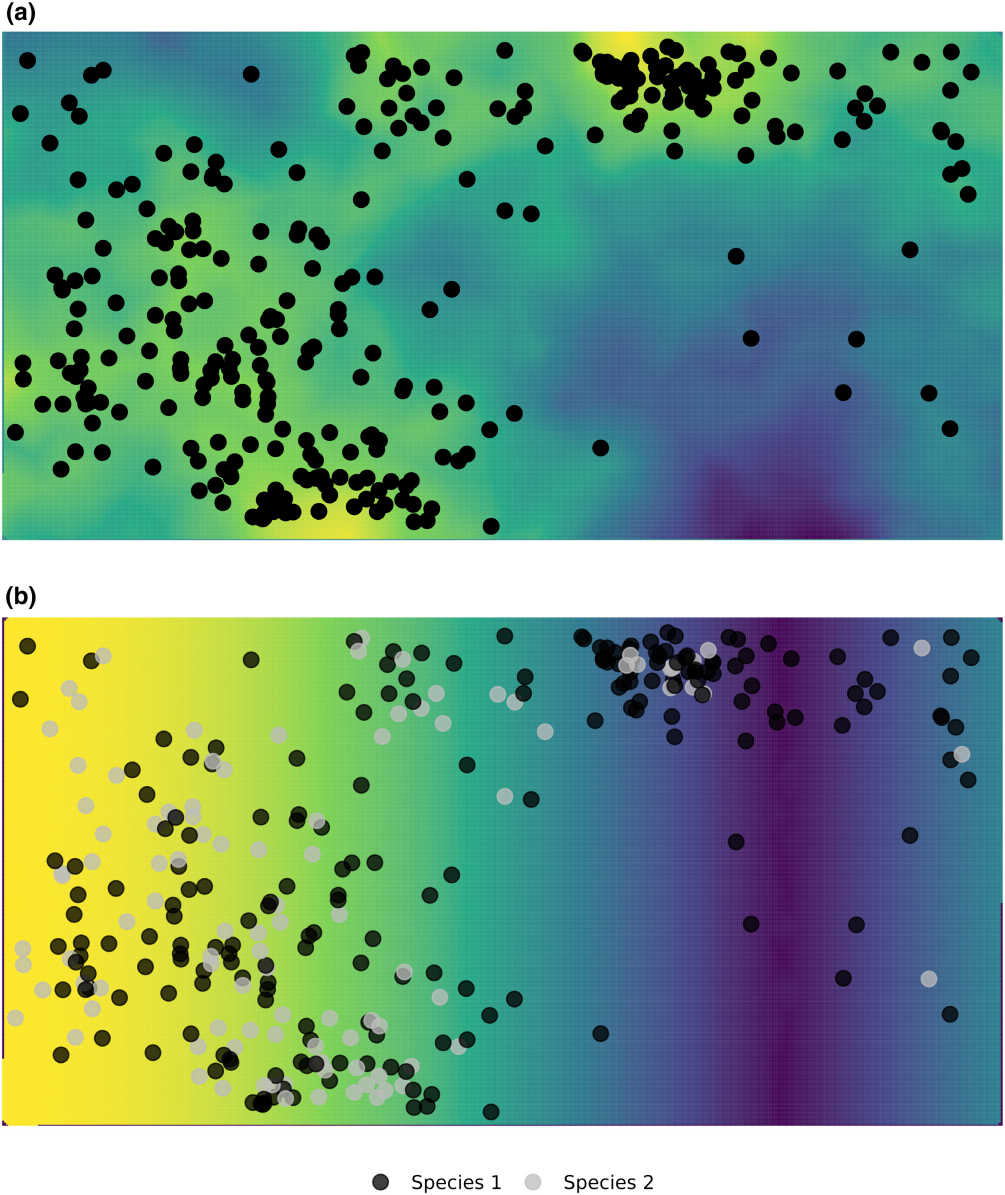
Plot (a) LGCP where the points are a realisation of an inhomogeneous Poisson process conditionally independent of the latent GRF. Parameters of the GRF control the expected spatial patterning of the points (i.e., the degree of clustering). Higher values of the GRF (here in yellow) gives rise to more clustered points, whereas cooler areas (here in blue) give rise to sparsely distributed points. Plot (b) A marked version of the LGCP shown in plot (a) where the chance of a Species 2 (grey) depends on the latent Gaussian intensity process in plot (a) as well as the surface in plot (b). That is, we are more likely to observe Species 2 where (1) there are more points, a process governed by the intensity surface in plot (a), and (2) the further to the left we are, this governed by the mark surface shown in plot (b).

Figure 2 plot (a) shows a realisation of a LGCP overlain on the simulated Gaussian random intensity process. Here, larger values of the field are yellow, these higher values gives rise to more clustered points; whereas cooler areas (i.e., blue) give rise to more sparsely distributed points. In practice, it is only the points we observe and from their distribution we estimate the assumed latent GRF.

Mathematically, a LGCP is a specific type of Cox process (Cox & Isham, 1980) based on a stationary and isotropic Gaussian random field, $G\left(x\right)$, with mean μ, variance–covariance matrix $Q^{-1}$ and covariance function $C_{Z}$, such that the random intensity surface is given by

(3) $$\Lambda \left(s\right)=\exp\left(\mathrm{X\beta}+G\left(s\right)\right)$$

for design matrix X and coefficients β (Ripley, 1981). It is conventional to use the Matérn covariance function to define the covariance of the random field, which has two parameters ψ and κ. Typically, functions of ψ and κ are reported as the spatial range $r=\frac{\sqrt{8}}{\kappa }$ and standard deviation $\sigma =\frac{1}{\sqrt{4{\pi \kappa }^{2}\psi^{2}}}$. Under INLA methodology the *practical* range is defined as the distance such that the correlation is ∼0.139 (Krainski et al., 2018). This range is given as $r\sqrt{8\nu }=\frac{\sqrt{8\nu }}{\kappa }$ where r is the spatial range (as above) and ν is the smoothness parameter, which can be thought of as controlling the degree of change (spikiness) in the field.

As $\Lambda \left(x\right)$, above, is unobserved, the evaluation of the model likelihood involves integration over the infinite‐dimensional distribution of $\Lambda \left(x\right)$, which typically results in a huge computational expense. An elegant solution is based on approximating the continuously indexed Gaussian field by a GMRF Rue and Tjelmeland (2002). More recently, this approach became particularly practical when Lindgren et al. (2011) derived a solution based on a Stochastic Partial Differential Equation (SPDE) whose solution is a Gaussian field with Matérn correlation. They found that, for certain parameter values, it was possible to represent this Gaussian Field by a GMRF, by using the Finite Element Method to provide a solution to a SPDE. To make the GMRF approximation valid, ν is fixed as $\alpha =\nu +\frac{d}{2}$, with α=2 (as Krainski et al. (2018)) in 2D (d=2), therefore ν=1. We use this approach in stelfi to approximate the assumed Gaussian random fields. This requires the construction of a Delaunay triangulation for the spatial domain, which forms the basis of the approximation. For this, stelfi uses exported functionality from INLA (Lindgren & Rue, 2015).

A spatiotemporal LGCP model, typically, uses auto‐regressive temporal dependence with an arbitrary number of time knots, i=1,…,n (e.g., month index):

$$\Lambda_{i}\left(s\right)=\exp\left(\mathrm{X\beta}+G_{i}\left(s\right)+\varepsilon \right)$$

Here $\Lambda_{i}\left(s\right)$ is the field intensity at time knot i and $G_{i}\left(s\right)$ the GMRF at the same time knot. Each $G_{i}\left(s\right)$ shares common values for ψ and κ, and successive random fields are correlated following $G_{i}\left(s\right)=\rho G_{i-1}\left(s\right)+\varepsilon_{i}$, where $\rho \in \left[-1,1\right]$.

### 2.4 A marked log‐Gaussian Cox process

Each point in a spatial pattern may have one or more associated marks (characteristics). In that case, we are interested not only in the spatial intensity of the points but also in the spatial distribution of the marks (e.g., species) and the dependence between the marks and the spatial intensity. Here, we can again assume a log‐Gaussian Cox model for the point pattern (as in the previous section), but also jointly model the marks. This is a useful model when the marks are not independent of clusters (e.g., certain species of shrub may be more likely to cluster together), and yet we are interested in the mark distribution independently of the inter‐point dependence.

Figure 2 plot (b) shows a realisation of a marked LGCP where the colours indicate the species of each point, where points could be animals or plants etc. In Figure 2, the chance of a Species 2 (grey) depends on the latent Gaussian intensity process in plot (a) as well as the mark‐specific surface in plot (b). This means that we are more likely to observe Species 2 where there are more points, a process governed by the GRF, and the further towards the left we are, this governed by the mark‐specific surface.

A marked LGCP can be written as an extension to Equation (3), where

(4) $$\begin{array}{r} \Lambda \left(s\right)=\exp\left(\mathrm{X\beta}+G\left(s\right)+\varepsilon \right),\\ M_{j}\left(s\right)=f^{-1}\left({\left(\mathrm{X\beta}\right)}_{m_{j}}+G_{m_{j}}\left(s\right)+\alpha_{m_{j}}\;G\left(s\right)+\varepsilon_{m_{j}}\right). \end{array}$$

Here, $\alpha_{m_{j}}$ are coefficient(s) linking the point process and the $n_{\text{mark}}$ mark(s), $m_{j}$ $\left(j=1,\ldots ,n_{\text{mark}}\right)$. The form of $M_{j}\left(s\right)$ and $f^{-1}$ depends on the assumed distribution of the marks. This joint model can be likened to the preferential sampling model proposed by Diggle and Su (2010) where the log‐Gaussian Cox model assumed for the point pattern is modelled jointly alongside the response. The response referred to in the preferential sampling model are, in the model above, the marks. The options available for the mark distribution in stelfi are given below.

1. If $m_{j}∼\text{Normal}\left(\mu_{j}\left(s\right),\sigma_{j}\right)$ then $M_{j}\left(s\right)=\mu_{j}\left(s\right)$ and $f^{-1}=I$ (user must supply the standard deviation $\sigma_{j}$),
2. If $m_{j}∼\text{Poisson}\left(\Lambda_{j}\left(s\right)\right)$ then $M_{j}\left(s\right)=\Lambda_{j}\left(s\right)$ and $f^{-1}=\exp$,
3. If $m_{j}∼\text{Binomial}\left(n_{j},p_{j}\left(s\right)\right)$ then $M_{j}\left(s\right)=p_{j}\left(s\right)$ and $f^{-1}=\text{logit}$ (user must supply the number of trials $n_{j}$), and
4. If $m_{j}∼\text{Gamma}\left({\text{shape}}_{j}\left(s\right),{\text{scale}}_{j}\right)$ then $M_{j}\left(s\right)={\text{shape}}_{j}\left(s\right)$ and $f^{-1}=\log$ (user must supply the log of the scale parameter $\log\left({\text{scale}}_{j}\right)$).

### 2.5 A spatiotemporal self‐exciting process

In this section we introduce a spatiotemporal self‐exciting process, which combines some of the models outlined in the previous sections. Such a model would enable the degree of spatiotemporal self‐excitement (e.g., that an event immediately increases of a future event in nearby space) to be inferred in addition to the effect of spatial and temporal covariates.

The Hawkes process is traditionally formulated as a temporal point process; however, we can also extend this to a spatiotemporal version. The spatiotemporal Hawkes processes fitted by stelfi have the temporal self‐excitation following an exponential decay function. The self‐excitation over space follows a Gaussian distribution centred on the triggering event. There are two formulations of this model. The default is that the Gaussian function has a fixed covariance matrix, independent of time. Alternatively, the covariance can be directly proportional to time, meaning that the self‐excitement radiates from the centre over time. This can be appropriate when the mechanism causing self‐excitement travels over space; however, it is very memory‐intensive. The spatiotemporal intensity function defined in stelfi is given by

(5) $$\lambda \left(s,t\right)=\mu +G\left(s\right)+\alpha \sum_{i:\tau_{i}<t}\left(\exp\left(-\beta^{*}\left(t-\tau_{i}\right)\right)K_{i}\left(s-x_{i},t-\tau_{i}\right)\right)+\varepsilon .$$

where μ is the background rate, β is the rate of temporal decay, α is the increase in intensity after an event, $\tau_{i}$ are the event times, and $x_{i}$ are the event locations (in 2D Euclidean space). The error term is given by ε. As in Equation (3) $G\left(s\right)$ is a Gaussian random field, the inclusion of which is optional. The spatial self‐excitement kernel is given by $K_{i}\left(s-x_{i},t-\tau_{i}\right)∼\text{Normal}\left(0,Q^{-1}\right)$; this can either be time‐independent where $Q^{-1}=\left[\begin{array}{cc} \sigma_{x}^{2} & {\rho \sigma }_{x}\sigma_{y}\\ {\rho \sigma }_{x}\sigma_{y} & \sigma_{y}^{2} \end{array}\right]$ or time‐dependent where $Q^{-1}=\left[\begin{array}{cc} \sigma_{x}^{2} & {\rho \sigma }_{x}\sigma_{y}\\ {\rho \sigma }_{x}\sigma_{y} & \sigma_{y}^{2} \end{array}\right]\times \left(t_{j}-t_{i}\right)$ for $t_{j}>t_{i}$.

### 2.6 Example

The BFRO is an organisation dedicated to investigating mystery of Sasquatch (bigfoot) (BFRO, 1995). The organisation collects reports from the general public detailing (supposed) Sasquatch sightings. Alongside a brief description of the incident each sighting occurrence is geocoded (latitude and longitude) and has a timestamp, with resolution varying depending on report. Although the existence of Sasquatch is unlikely, the structure of the data is akin to many other citizen science datasets. In addition, the questions and issues that arise from such data are equivalent to those ecologists have in dealing with sightings data for non‐mythical creatures. For example, sightings often cluster in time and space due to a variety of reasons. These reasons may be environmental, ecological or simply be an artefact of the observation process (e.g., people are more likely to visit areas where a certain species has previously been sighted and are therefore more likely to observe it). The interest is often to tease apart or account for the multiple reasons behind the clusters (or otherwise) of sightings. In this section, we illustrate the fitting of the most common models outlined in the sections above using data collated by Renner (2021) obtained from the BFRO containing the temporal and spatial locations of claimed sightings of Sasquatch across the contiguous USA from 2000 to 2006. All the required R code for the analysis summarised in this section as well as more detail about the Sasquatch data is given in Appendix S1 as well as the package website (https://cmjt.github.io/stelfi/). In Appendix 1, we show via simulation the performance of the model fitting functions described below.

### 2.7 Self‐exciting sightings using fit_hawkes()

**FIGURE 3:**
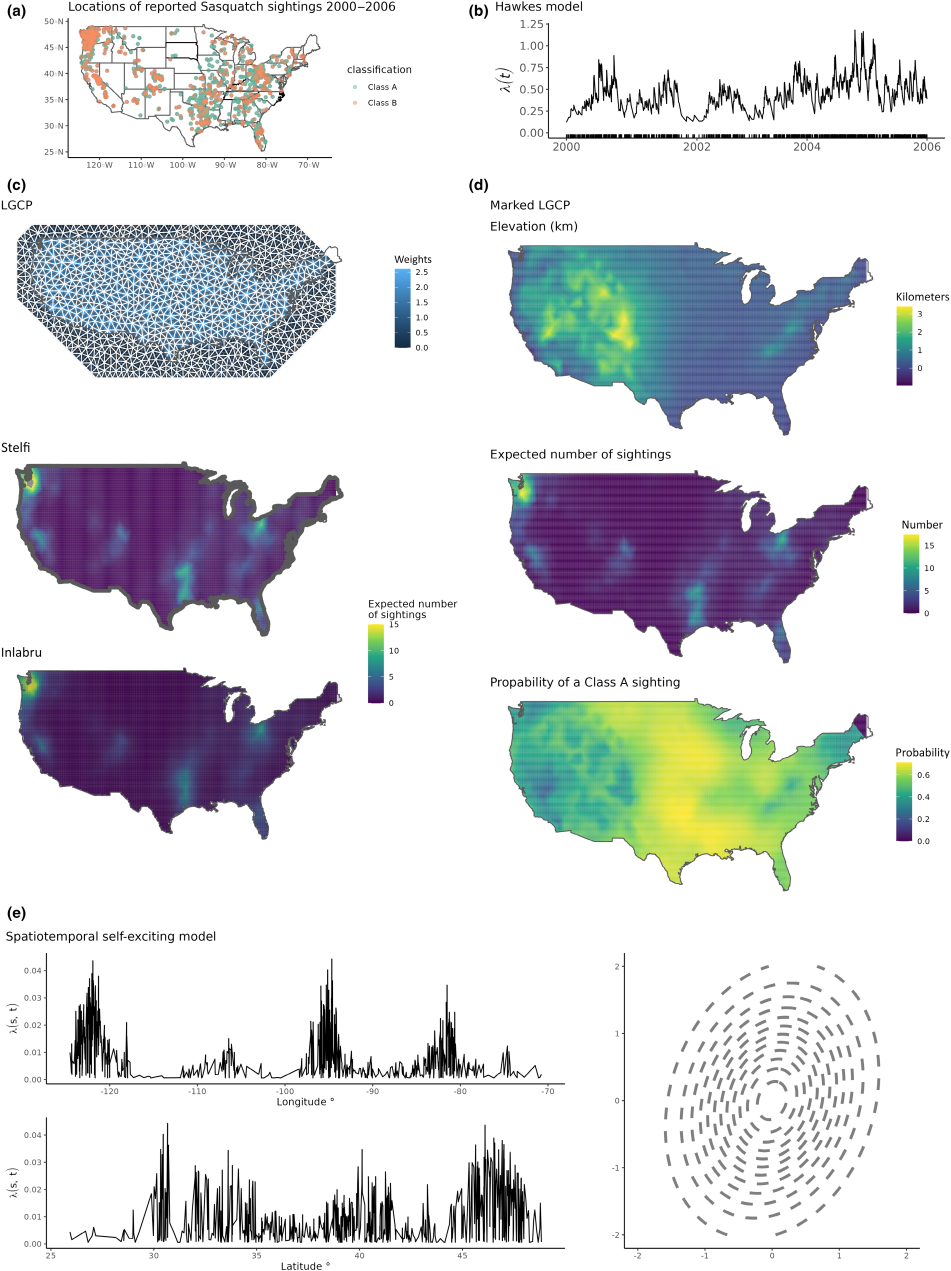
Plot (a) shows the observed point pattern, points are coloured by marks. Plot (b) shows the fitted Hawkes intensity (black line) given by Equation (1) to the temporal point pattern (rug plot) of Sasquatch sighting times. Plot (c) shows the Delaunay triangulation (white) and the resulting Voronoi tessellation (grey)where each Voronoi cell is coloured according to the integration weight used in the model; In addition, the estimated intensity surface obtained by stelfi and for comparison inlabru are shown. Plot (d) shows the components of the marked LGCP given in Equation (6): the elevation covariate, the fitted intensity surface, and the estimated mark process. Plot (e) shows the spatiotemporal intensity, over Latitude and Longitude, given by Equation (5) and the estimated Gaussian kernel density for $Q^{-1}$ shown in Table 1.

From 2000 up until 2006 there were n=972 recorded Sasquatch sightings over T=2188 days in the contiguous USA, see plot (a) in Figure 3. These sightings data were recorded at a daily resolution; however, the Hawkes process assumes unique occurrence times. Therefore, to fit the model we jitter each observation slightly (by no more than 24 h). As the data cover such a long span this makes little difference in practice; however, in general, great care should be taken when including a jitter.

Hawkes processes are particularly useful tools in inferring the, potentially, contagious nature of clustered event‐type data. For example, we may be interested in inferring to what extent the claimed sighting of bigfoot incites another in the near future. To fit a Hawkes process given by Equation (1) using stelfi to the Sasquatch data we call.
fit_hawkes(times, parameters)

where times is a vector of Sasquatch sighting times (i.e., temporal locations as described above) and parameters is a vector of parameter starting values. An additional argument, marks is not supplied, which means by default $m\left(t\right)$ in Equation (1) is fixed at 1. The fitted model, estimated self‐exciting temporal intensity of sightings, can be seen in plot (a) Figure 3, where the rug plot on the *x*‐axis shows the temporal location of recorded sightings.

Table 1 compares the parameter estimates and standard errors returned by stelfi with emhawkes and hawkesbow (see the Appendix S1 for the required R code). From these estimated values, we estimate the expected background rate of sightings (i.e., independent sightings) as $\hat{\mu }T=0.12\times 2188∼263$, which indicates that ∼263 sightings were principal (independent) sightings and that the remaining were due to self‐excitement. The expected number of sightings “triggered” by anyone sighting is estimated as $\frac{\hat{\alpha }}{\hat{\beta }}=\frac{0.06}{0.09}=\frac{2}{3}$ and the expected number of descendants per sighting is estimated as $\frac{\beta }{\hat{\beta }-\hat{\alpha }}=\frac{0.09}{0.09-0.06}=3$. The rate of decay for the self‐excitement is estimated as $\frac{1}{\hat{\beta }}=\frac{1}{0.09}∼11$ days, indicating that after ∼11 days a sighting is likely independent of any historic sighting.

**TABLE 1:** Table showing the estimated parameter values and standard errors for the fitted models discussed.

Model	Parameter(s)	stelfi		emhawkes		hawkesbow		inlabru	
		Estimate	Std. error	Estimate	Std. error	Estimate	Std. error	Estimate	Std. error
Hawkes	μ	0.12	0.04	0.12	0.05	0.12	0.04	–	–
	α	0.06	0.03	0.07	0.03	–	–	–	–
	$\frac{\alpha }{\beta }$	–	–	–	–	0.73	0.10	–	–
	β	0.09	0.05	0.09	0.06	0.09	0.05	–	–
	log‐likelihood	−1681.156		−1676.068		−1681.156		–	
LGCP	$\beta_{0}$	−0.77	0.36	–	–	–	–	−0.49	0.25
	r	7.34	1.11	–	–	–	–	7.94	0.83
	σ	1.35	0.15	–	–	–	–	0.87	0.06
	system.time()$elapsed	25.37		–		–		114.83	
Marked LGCP	$\beta_{0}$	−0.823	0.358	–	–	–	–	–	–
	$\beta_{1}$	0.114	0.251	–	–	–	–	–	–
	$\beta_{0}^{m}$	0.227	0.284	–	–	–	–	–	–
	$\beta_{1}^{m}$	−0.372	0.178	–	–	–	–	–	–
Self‐exciting spatiotemporal model	$\alpha_{m}$	0.044	0.063	–	–	–	–	–	–
	μ	0.0006	0.0001	–	–	–	–	–	–
	α	0.0215	0.0028	–	–	–	–	–	–
	β	0.0215	0.0028	–	–	–	–	–	–
	$\sigma_{x}$	0.7060	0.0518	–	–	–	–	–	–
	$\sigma_{y}$	0.9132	0.0913	–	–	–	–	–	–
	ρ	0.1999	0.1226	–	–	–	–	–	–
	$Q^{-1}$	$\left[\begin{array}{cc} 0.498 & 0.129\\ 0.129 & 0.834 \end{array}\right]$		–		–		–	

*Note*: Hawkes: Estimated parameter and log‐likelihood values of a Hawkes process, given by Equation (1), modelling the reported temporal occurrence of Sasquatch sightings. These are shown for the packages stelfi, emhawkes and hawkesbow. Note that hawkesbow returns the estimate of $\frac{\alpha }{\beta }$ and the standard errors shown are calculated from the returned Hessian matrix H (i.e., $\sqrt{\mathrm{diag}\left({\left(-H\right)}^{-1}\right)}$). LGCP: Estimated intercept, $\beta_{0}$, and GMRF parameter values (range r and standard deviation σ) for the LGCP process (Equation 3) fitted to reported Sasquatch sighting locations. Estimates and standard errors are shown for both stelfi and inlabru along with the time elapsed to fit the models. Marked LGCP: Estimated parameter of the joint model given by Equation (6) fitted using stelfi::fit_mlgcp(). Self‐exciting spatiotemporal model: Estimated parameter values, standard errors, and the spatiotemporal precision matrix for the model given by Equation (5) fitted using stelfi::fit_stelfi().

Giving the fitted model object to the stelfi functions show_hawkes() and show_hawkes_GOF() will plot the fitted model and a range of goodness‐of‐fit plots, respectively. In addition, if return_values = TRUE is set, the transformed inter‐arrival times from the fitted models are returned, see the package website (https://cmjt.github.io/stelfi/) for examples.

### 2.8 Spatial distribution of Sasquatch sightings using fit_lgcp()

The n=972 Sasquatch sightings occurred within an area ($A∼7,771,155{\mathrm{km}}^{2}$) (the contiguous USA, plot (a) in Figure 3). This shows that sightings are, spatially, quite sparse at ∼0.000125 per ${\mathrm{km}}^{2}$ over the 5 years. Most sightings occur in Washington and northwest Oregon (to be expected as Bigfoot is purported to inhabit the wild and forested areas of Oregon and the West Coast of North America), with another dense spot near the Texas–Louisiana border.

To infer the spatial distribution of bigfoot (sightings) we fit a LGCP given by Equation (3) with X=1 and $\beta =\beta_{0}$ (i.e., an intercept‐only fixed effect) in stelfi via
fit_lgcp(locs, sf, smesh, parameters)

where locs is a data frame of 2D locations, sf a simple features polygon of the domain/window, smesh a Delaunay triangulation covering the domain, and parameters a vector of parameter starting values.

We compare the parameter estimates returned by stelfi to inlabru in Table 1 (see the Appendix S1 for the required R code). Both packages use the SPDE approach to estimate the latent field (Lindgren et al., 2011); however, stelfi uses a frequentist framework with custom‐written TMB templates and inlabru uses a Bayesian framework.

To estimate the total number of events, integration weights are required. These weights are the area surrounding the mesh nodes; the expected number of events at each node can be thought of as being proportional to the weights (Simpson et al., 2016). The weights used are the areas of the Voronoi cell centred on each mesh node (called the *dual mesh* in the INLA framework). The inbuilt get_weights() can be used in stelfi to extract the weights used given a model object and a Delaunay triangulation. To plot these weights set plot = TRUE, this results in the top figure of plot (c) in Figure 3, which shows the Delaunay triangulation (white) and the resulting Voronoi tessellation (grey). Each Voronoi cell is coloured according to the integration weight used in the model; note that all cells outwith the contiguous USA land mass are set to zero and therefore do not contribute to the model. Using these integration weights and the estimated parameters (Table 1) we estimate the total number of events using $\sum_{k=1}^{m}w_{k}\;\exp\left(\beta_{0}+g_{k}\right)$ were m are the number of mesh nodes, $w_{k}$ is the weight and $g_{k}$ the estimated mean of the random field at the kth mesh node; from this, we get an estimate of 970 events using stelfi and 987 using inlabru (observed n=972 sightings).

Figure 3 plot (c) shows the estimated intensity surface obtained by each package. To return the estimated values of the random field at each mesh node from stelfi we use get_fields(), providing the fitted model object, fit, and the mesh, smesh. There are additional options to return the estimated standard deviation of the field(s) with sd = TRUE and to plot the returned values with plot = TRUE. The estimated intensity surface (middle plot) is plotted using show_lambda with the additional arguments sf and clip = TRUE, to plot only the values within the domain. The plots are ggpot2 objects and therefore additional geom's can be added as layers. See the Appendix S1 for the required inlabru code for the comparisons given.

### 2.9 A marked LGCP using fit_mlgcp()

Categorical marks of ecological objects are commonplace (e.g., species, sex, classification) and often there is interest in inferring the point‐mark dependence and the mark‐specific distribution (i.e., independent of point location), see Figure 2. The Bigfoot Field Researchers Organisation (BFRO) (BFRO, 1995) categorise Sasquatch sightings into three classes: (1) Class A, clear; (2) Class B, observed at a great distance or in poor lighting conditions; and Class C, second‐ and third‐hand reports, or stories with an untraceable source.

To illustrate a marked LGCP we consider only Class A and Class B sightings (n=971) and jointly model Sasquatch sightings alongside the probability of that sighting being of Class A (clear). Doing so will allow us to infer the, potentially, unexplained process driving clear sightings (e.g., lack of foliage). To do this, we set $m_{i}=1$ if the sighting is Class A and $m_{i}=0$ if the sighting is Class B for i=1,…,971. The joint model we fit is a special case of Equation (4):

(6) $$\begin{array}{r} \Lambda \left(s\right)=\exp\left(\beta_{0}+\beta_{1}x_{\text{elev}}\left(s\right)+G\left(s\right)+\varepsilon \right)\\ \text{logit}{\left(p\left(s\right)\right)}^{-1}=\beta_{0^{m}}+\beta_{1}^{m}x_{\text{elev}}\left(s\right)+G_{m}\left(s\right)+\alpha_{m}\;G\left(s\right)+\varepsilon_{m} \end{array}$$

where $m\left(s\right)∼\text{Bernoulli}\left(p\left(s\right)\right)$ and the spatial intensity of all sightings is, as previously, $\Lambda \left(s\right)$. A spatial covariate, $x_{\text{elev}}\left(s\right)$ the elevation in kilometres obtained from the R package elevatr (Hollister et al., 2021), is included in each linear predictor (see plot (d) in Figure 3). Different coefficients are estimated for the LGCP and the mark process ($\beta_{1}$ and $\beta_{1}^{m}$ respectively). It is a reasonable supposition that elevation might affect the clarity of the sighting as well as their number and therefore it is included, separately, as a covariate in both linear predictors. The shared GMRF $G\left(s\right)$ represents the spatial autocorrelation of the sighing locations, which may also contribute to the mark process according to the estimated parameter $\alpha_{m}$. The GMRF $G_{m}\left(x\right)$ is unique to the mark and may be thought of as representing any spatial autocorrelation not explained by the point locations; this interpretation is somewhat ad hoc however and care should be taken.

To fit this model in stelfi, we use
fit_mlgcp(locs, marks, sf, smesh, parameters, methods = 2, fields = 1, covariates = covariates, pp_covariates = 1, marks_covariates = 1)

where, in addition to the arguments we supply to fit_lgcp() we set methods = 2 (Binomial distribution assumed for the marks with the number of trials =1, Bernoulli), and fields = 1 (to include mark‐specific random field). We also supply a vector marks, the mark values at each location and covariates the elevation in kilometres at each mesh node obtained from the R package elevatr (Hollister et al., 2021). Setting pp_covariates = 1 and marks_covariates = 1 means that the first column of covariates will be included in the linear predictor for both the LGCP and the mark.

Table 1 gives the estimated parameter values. The magnitude of the contribution of the shared random field to the mark is given by the parameter $\alpha_{m}$, which is estimated as 0.044 $\left(\text{standard error}\;0.063\right)$. This indicates that there is no point‐mark dependence because the estimated standard error is greater than the distance between the estimated coefficient value and zero. That is, the probability of a Class A sighting does not seem to change in relation to the spatial intensity of sightings. The mark‐specific elevation coefficient is estimated as −0.372 $\left(\text{standard error}\;0.178\right)$ indicating that the probability of a Class A sighting (slightly) decreases as elevation increases. This seems reasonable, people are perhaps less likely to get a clear sighting the greater the elevation due to rougher terrain etc. Looking at the estimated mark process in Figure 3 plot (d) we see that the probability of a Class A sighting is greatest in the eastern states. The elevation coefficient for the LGCP is estimated as 0.113 $\left(\text{standard error}\;0.251\right)$ indicating that sightings increase as elevation increases. Again, this seems reasonable, sightings are more prolific the greater the elevation/the more remote the terrain, see Figure 3 plot (d).

### 2.10 Self‐exciting spatiotemporal sightings using fit_stelfi()

Fitting a spatiotemporal self‐exciting model requires that the times of the events be unique, therefore, we have removed duplicated daily sightings for the Sasquatch data. Not something that is advised in practice. However, in the interest of demonstration, we do so here resulting in 677 sightings on unique days; see the Appendix S1 for the required R code.

To fit a time‐independent model as Equation (5) we use the stelfi function
fit_stelfi(times, locs, sf, smesh, parameters)

where the arguments are as above. In addition to these typical arguments, there are two logical arguments to fit_stelfi that control the form of spatiotemporal self‐excitement. Setting time_independent = TRUE (default FALSE) makes the self‐excitement kernel time‐dependent, see the package website for more details (https://cmjt.github.io/stelfi/). Setting GMRF = TRUE (default FALSE) adds a Gaussian Markov random field onto the baseline spatiotemporal intensity.

Parameter estimates for the fitted model are given in Table 1. Figure 3 plot (e) show the spatiotemporal intensity, over Latitude and Longitude, as well as the bivariate Gaussian density with $Q^{-1}$ as shown in Table 1. From this, we can see that the estimated spatiotemporal intensity over longitude shows spikes of sightings clustered around 120° W, 95° W and 80° W, correspondingly over latitude the intensity is relatively similar, however, 30° N and 45° N seem to have a greater intensity. These locations correspond to Washington, Texas and Ohio, matching the observed data structure over those states. The contour plot shows the estimated Gaussian spatial kernel in Equation (5); from the elliptical shape this shows that the spatial dependency is more dispersed across latitude (*y*‐axis) than longitude (*x*‐axis).

## 3 CONCLUSIONS

The R package stelfi offers the functionality to fit a variety of Hawkes and log‐Gaussian Cox process models via TMB (Kristensen et al., 2016). The package is available on CRAN, with the development version on GitHub (https://github.com/cmjt/stelfi). The package version (v1.0.1) used in this manuscript has the persistent identifier https://doi.org/10.5281/zenodo.10515700.

Modelling point pattern data is a huge part of ecological and environmental statistics. The novelty of the package is twofold (1) custom‐written C++ templates take advantage of TMB, fitting models via maximum likelihood; (2) it facilitates the fitting of temporal and spatiotemporal self‐exciting point process models.

We demonstrate the core functionality of stelfi using Sasquatch sightings data. These data are used for illustration purposes only, yet the structure is typical of data often seen in ecology and environmental science. Where possible, we demonstrate the performance of stelfi compared to the R packages that fit similar models via different means.

This package offers a much‐needed addition to the point process field. It provides an alternative to existing model fitting frameworks and includes functionality that extends the currently available methods. We foresee the package becoming requisite in the fields of ecological and environmental modelling and beyond.

## AUTHOR CONTRIBUTIONS

**Charlotte M. Jones‐Todd:** Conceptualization (lead); formal analysis (lead); funding acquisition (lead); methodology (lead); project administration (lead); software (lead); writing – original draft (lead). **Alec B. M. van Helsdingen:** Software (supporting); writing – review and editing (supporting).

## FUNDING INFORMATION

This work was supported by Marsden Fund proposal UOA 3723517 and Asian Office of Aerospace Research & Development grant FA2386‐21‐1‐4028.

## CONFLICT OF INTEREST STATEMENT

The authors have no conflicts of interest.

## Supporting information


Appendix S1.

## Figures and Tables

**FIGURE A1:**
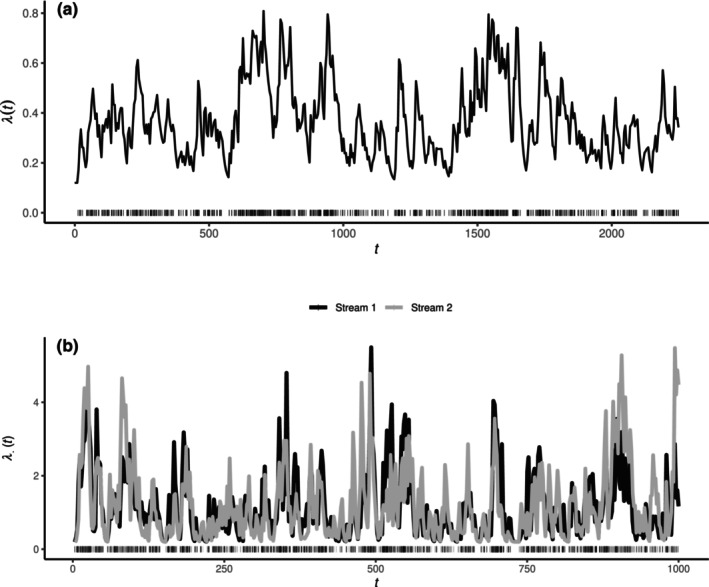
(a) Single realisation of a simulated temporal Hawkes process (Equation 1) with μ=0.12, α=0.06 and β=0.09. The realisation is simulated over T=2500 days, similar to the time frame the Sasquatch data covers. The corresponding Hawkes intensity, $\lambda \left(t\right)$, is shown by the solid line and each event is denoted by ∣. (b) A single realisation of a simulated temporal bivariate Hawkes process (Equation (2) where N=2) with $\mu =\left(0.2,0.2\right)$, $\alpha =\left(\begin{array}{cc} 0.5 & 0.1\\ 0.1 & 0.5 \end{array}\right)$, and $\beta =\left(0.7,0.7\right)$. The Hawkes intensities are shown by the solid lines and each event is denoted by ∣. The colours of the lines and the events indicate the two different streams.

**FIGURE A2:**
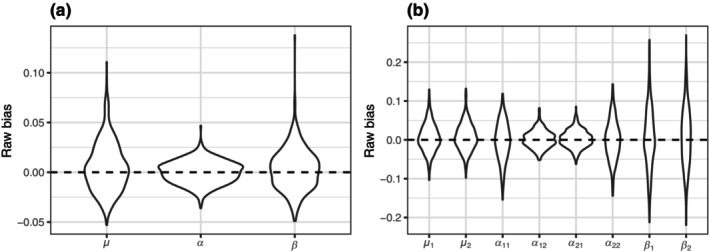
Raw bias (i.e., for any parameter value θ, raw bias = $\hat{\theta }-\theta$) for each simulation study (a) a temporal Hawkes process and (b) a bivariate temporal Hawkes process. Table A1 gives the parameter values used in the simulations as well as the percentage bias in each case.

**FIGURE A3:**
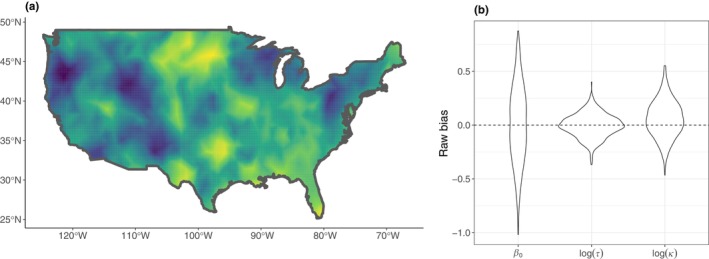
(a) Single realisation of a simulated spatial GMRF over the contiguous USA, with parameter values β=−0.76, $\log\left(\tau \right)=-0.60$ and $\log\left(\kappa \right)=-0.95$ (i.e., r=7.34 and σ=1.34, values estimated in the corresponding Sasquatch model). (b) The raw bias (i.e., for any parameter value θ, raw bias = $\hat{\theta }-\theta$) for each parameter of the LGCP.

**FIGURE A4:**
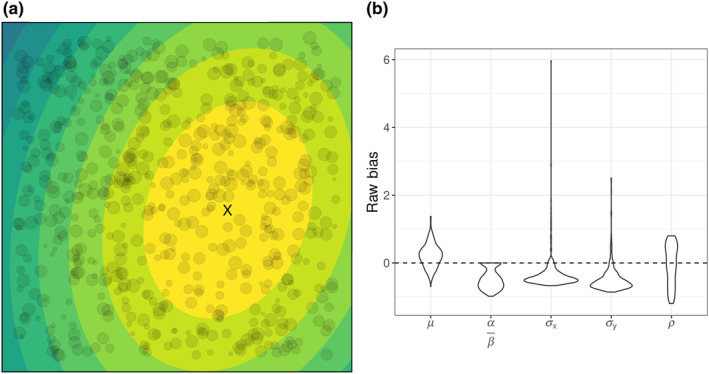
(a) Single realisation of a spatiotemporal Hawkes model (Equation 5) where the spatial self‐excitement follows a bivariate Gaussian kernel with $\sigma_{x}=0.7$, $\sigma_{y}=0.9$ and ρ=0.2. The timestamp of each point is denoted by the size of the plotting character (the larger the later the timestamp). Given an arbitrary point, X, the bivariate Gaussian kernel corresponding to the spatial self‐excitation is shown by the coloured contours centred at this point. (b) The raw bias (i.e., for any parameter value θ, raw bias = $\hat{\theta }-\theta$) for each parameter of the spatiotemporal Hawkes model.

## Data Availability

The data used in this manuscript are shipped with the R package stelfi available from the Comprehensive R Archive Network (CRAN) (https://cran.r‐project.org/package=stelfi). Furthermore, the R package code is open source and available from the GitHub repository https://github.com/cmjt/stelfi (https://doi.org/10.5281/zenodo.10515700). All model fitting code and package version information etc. is given in Appendix S1, https://doi.org/10.5281/zenodo.10516195.

## APPENDIX 1 SIMULATION STUDY

In this section, we show via simulation the performance of the model fitting functions described in the manuscript. In each case, we simulate 500 realisations of a temporal/spatial/spatiotemporal point process using, where applicable, the same parameter values estimated from fitting the model to the Sasquatch data. We use these parameter values so that the simulated data are a similar structure to the Sasquatch data modelled. We then fit the same (i.e., known true) model to each realisation and assess model performance using the estimated parameters form the fitted models.

Plot (a) in Figure A1 shows a single realisation of a temporal Hawkes process (Equation 1) with μ=0.12, α=0.06, and β=0.09 over a period of T=2500 days (similar to the time frame the Sasquatch data covers). Plot (b) in Figure A1 shows a single realisation of a simulated temporal bivariate Hawkes process (Equation (2) where N=2) with $\mu =\left(0.2,0.2\right)$, $\alpha =\left(\begin{array}{cc} 0.5 & 0.1\\ 0.1 & 0.5 \end{array}\right)$, and $\beta =\left(0.7,0.7\right)$. Here event type 1 (Stream 1) is shown in black and event type 2 (Stream 2) is shown in black. Figure A2 shows the raw bias (i.e., for any parameter value θ, raw bias = $\hat{\theta }-\theta$) for the parameters of (a) a temporal Hawkes process and (b) a bivariate temporal Hawkes process. A total of 500 realisations are simulated and then the same model fitted to each realisation; Table A1 gives the parameter values used in the simulation as well as the percentage bias ($\bar{\hat{\theta }}-\theta /\theta$, where $\bar{\hat{\theta }}$ is the average estimated parameter value across all fitted models) for each simulation.

**TABLE A1:** True parameter values and the calculated percentage bias ($\bar{\hat{\theta }}-\theta /\theta$, where $\bar{\hat{\theta }}$ is the average estimated parameter value across all fitted models) for each simulation study.

Model	Parameter	Value	% Bias
Hawkes	μ	0.12	4.42
	α	0.06	0.18
	β	0.09	3.60
Multi. Hawkes	μ	$\left(0.2,0.2\right)$	$\left(2.78,3.55\right)$
	α	$\left[\begin{array}{cc} 0.5 & 0.1\\ 0.1 & 0.5 \end{array}\right]$	$\left[\begin{array}{cc} -0.73 & 1.63\\ 2.59 & 0.03 \end{array}\right]$
	β	$\left(0.7,0.7\right)$	$\left(0.68,1.08\right)$
LGCP	β	−0.76	−0.78
	$\log\left(\tau \right)$	−0.60	0.87
	$\log\left(\kappa \right)$	−0.95	−6.05
Self‐exciting spatiotemporal model	μ	2	10
	$\frac{\alpha }{\beta }$	1	−46
	$\sigma_{x}$	0.7	−49
	$\sigma_{y}$	0.9	−53
	ρ	0.2	−35

*Note*: In each case, a total of 500 realisations of the corresponding point process were simulated and then the same (i.e., known true) model fitted to each realisation.

Plot (a) in Figure A3 shows a single realisation of a simulated spatial GMRF (see Equation 3) over the contiguous USA, with parameter values β=−0.76, $\log\left(\tau \right)=-0.60$ and $\log\left(\kappa \right)=-0.95$ (i.e., r=7.34 and σ=1.34, values estimated in the corresponding Sasquatch model). During the simulation study 500 realisations of a LGCP (Equation 3) were simulated with the parameter values given in Table A1 and the same model (i.e., known true) fitted to each realisation. Plot (b) in Figure A3 shows the raw bias for each parameter.

Plot (a) in Figure A4 shows a single realisation of a simulated spatiotemporal Hawkes process (Equation 5) where the timestamp of each point is denoted by the size of the plotting character (the larger the later the timestamp). An arbitrary point in the pattern is denoted X and the bivariate Gaussian kernel corresponding to the spatial self‐excitation is shown by the coloured contours (i.e., a greater chance of a point in the near future in the lighter‐coloured areas than the darker ones). The Gaussian kernel has parameter values $\sigma_{x}=0.7$, $\sigma_{y}=0.9$ and ρ=0.2 corresponding to the estimated values from the fitted Sasquatch model. As previously, 500 realisations of the process were simulated with the parameter values given in Table A1 and the same model fitted to each realisation. Plot (b) in Figure A4 shows the raw bias for each parameter with the % bias shown in Table A1.

From plot (b) in Figure A4 and Table A1 we can see clear negative bias in the estimation of the self‐excitement parameters. This is likely due to the weak spatial self‐excitement process (i.e., relatively small values of α, $\sigma_{x}$, $\sigma_{y}$ and ρ) inherent in the simulated data. This is evident in the coloured contours of plot (a) in Figure A4, which are relatively smooth and decay slowly over space.
