# Supplementary material for: stelfi: An R package for fitting Hawkes and log‐Gaussian Cox point process models
Source: Ecol Evol. 2024 Feb 13;14(2):e11005. doi: 10.1002/ece3.11005 (PMC10864691; doi:10.1002/ece3.11005)
Supplement: Supplementary file 1 — Appendix S1. [file ECE3-14-e11005-s001.zip › codebook_stelfi_ecology_and_evolution.pdf]

Supplementary material for ‘stelfi: An R package for fitting Hawkes and log-Gaussian Cox point process models’.

Supplementary material

A guide to the analysis summarised in the **Example** section of the manuscript along with all required R code.

Package information

All required packages (and versions) used are listed below. Please use `install.packages(...)` to install from CRAN unless stated otherwise.

```
##############
## packages ##
##############
## package being showcased
library(stelfi)
## spatial data manipulation
library(sf)
## country maps
library(maps)
## plotting & pipes & data wrangling
library(tidyverse)
## packages to compare Hawkes process fit
library(emhawkes)
library(hawkesbow)
library(hawkes)
## packages to compare LGCP model fit
library(inlabru)
## required non-CRAN package for inlabru
## use install.packages("INLA",repos=c(getOption("repos"),
## INLA="https://inla.r-inla-download.org/R/stable"), dep=TRUE)
## to install
library(INLA)
##################
## version info ##
##################
R.version.string
```

```
## [1] "R version 4.3.1 (2023-06-16)"
```

```
Sys.info()['sysname']
```

```
## sysname
## "Linux"
```

```
pkgs <- c("stelfi", "sf", "maps",  "tidyverse",
          "emhawkes", "hawkesbow", "hawkes",
          "inlabru", "INLA")
```

```
sapply(pkgs, packageVersion)
```

```
## $stelfi
## [1] 1 0 1
##
## $sf
## [1]  1  0 14
##
## $maps
## [1] 3 4 1 1
##
## $tidyverse
## [1] 2 0 0
##
## $emhawkes
## [1] 0 9 7
##
## $hawkesbow
## [1] 1 0 2
##
## $hawkes
## [1] 0 0 4
##
## $inlabru
## [1] 2 9 0
##
## $INLA
## [1] 23  4 24
```

Sasquatch data

```
################################
## data shipped with package ##
################################
data("sasquatch", package = "stelfi")
```

```
## needed for GDAL shipped with older Ubuntu dist
sf::st_crs(sasquatch) <- "+proj=longlat +datum=WGS84 +no_defs +ellps=WGS84 +towgs84=0,0,0"
p1 <- ggplot(sasquatch, aes(x = date)) + geom_histogram(bins = 200) +
  ylab("Frequency") + xlab("") +
  theme_classic() + geom_rug(alpha = 0.4)
p1 + ggtitle("a)")
```

```
us <- maps::map("state", fill = TRUE, plot = FALSE) %>%
    sf::st_as_sf() %>%
    sf::st_make_valid()
locations <- ggplot(sasquatch) +
  geom_sf(alpha = 0.3) +
  coord_sf() +
  geom_sf(data = us, fill = NA) +
  theme_classic() + ggtitle("Locations of reported Sasquatch sightings 2000 to 2006")
locations
```

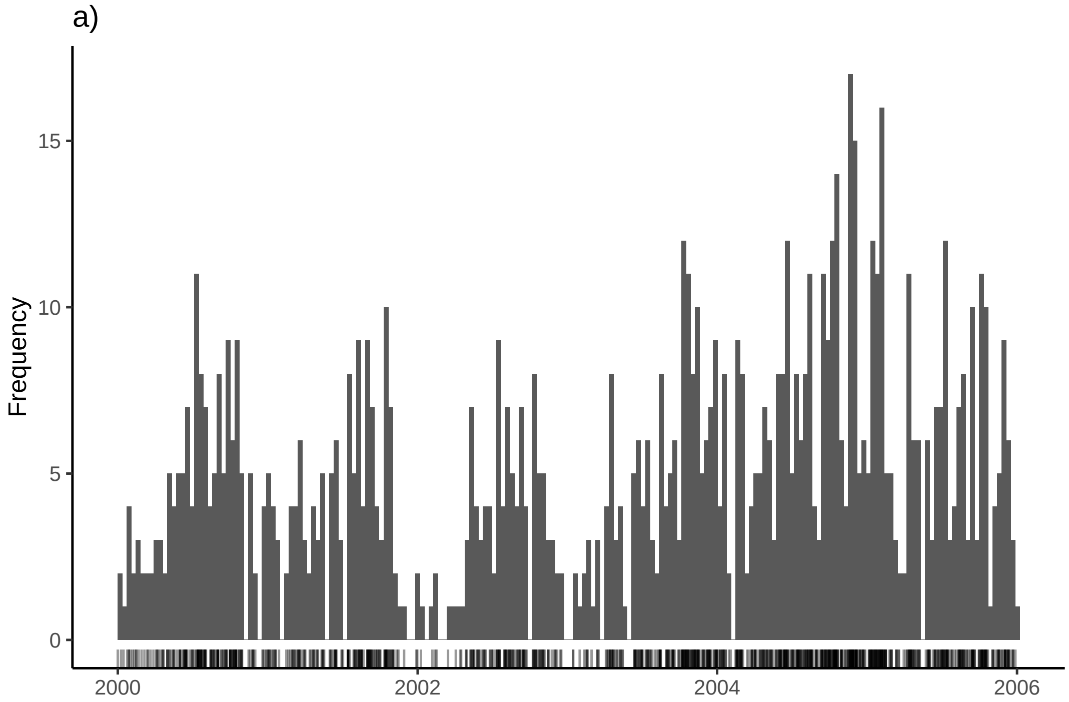

Figure 1: Reported Sasquatch sightings 2000–2006 a) frequency of events, and b) reported location of sighting.

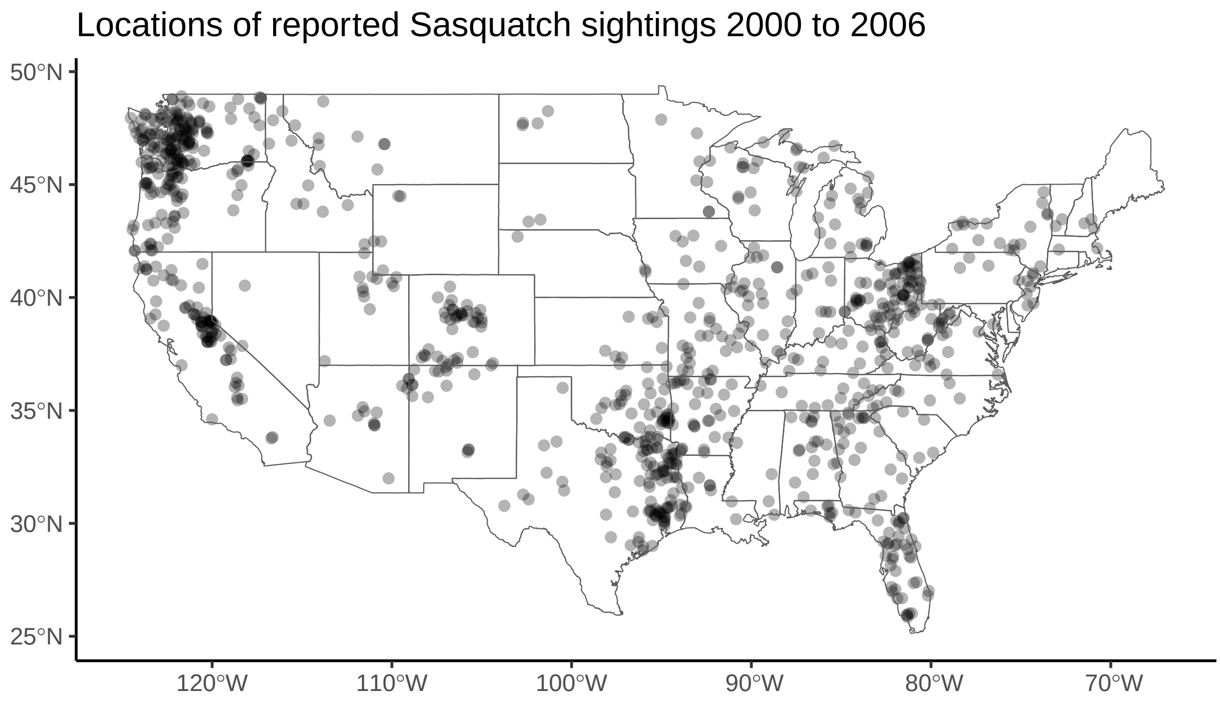

Figure 2: Reported Sasquatch sightings 2000–2006 a) frequency of events, and b) reported location of sighting.

Fitting a temporal Hawkes model to the unique sighting times

```
## data prep
set.seed(1234)
## times must be unique and in ascending order
times <- sasquatch %>%
  mutate(time = difftime(date, min(date), units = "days")) %>%
  mutate(time = time + runif(nrow(sasquatch),0, 1)) %>%
  mutate(time = time - min(time)) %>%
  sf::st_drop_geometry() %>%
  select(time) %>%
  unlist() %>%
  as.numeric() %>%
  sort()
```

Using stelfi

```
## parameter starting values
params <- c(mu = 9, alpha = 0.3, beta = 1)
## fit model
fit <- fit_hawkes(times = times, parameters = params)
## estimated parameter values
## shown in Table 3
get_coefs(fit)
```

```
##          Estimate Std. Error
## mu    0.12189479 0.04132070
## alpha 0.06623179 0.03034511
## beta  0.09075581 0.05092202
```

```
## negative log-likelihood
## shown in Table 3
fit$objective
```

```
## [1] 1681.156
```

Functions to plot fitted model and assess goodness-of-fit.

```
## plot fitted model
show_hawkes(fit)
```

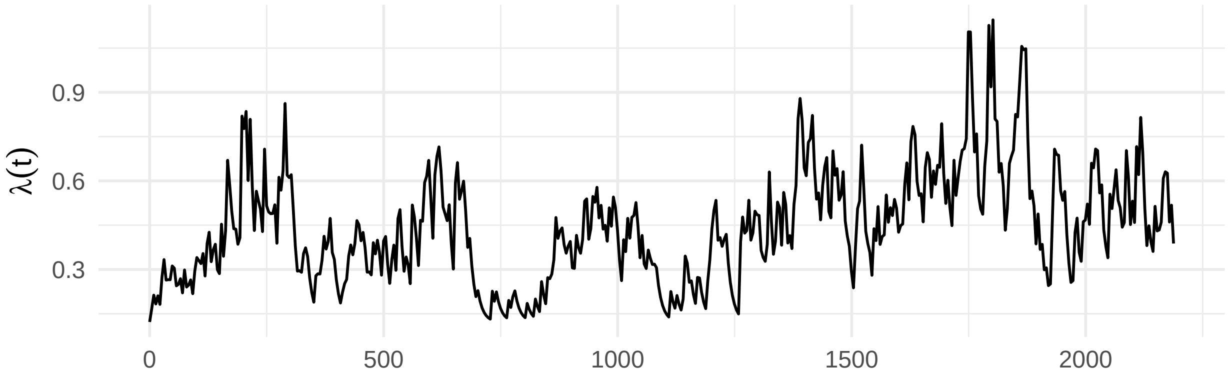

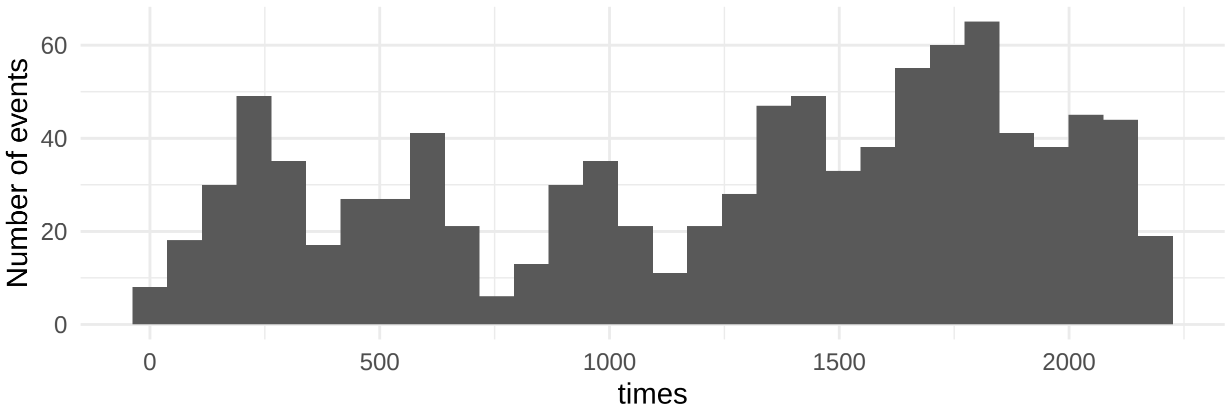


```
## plot and print out goodness-of-fit measures
show_hawkes_GOF(fit)
```

```
##
##  Asymptotic one-sample Kolmogorov-Smirnov test
##
## data:  interarrivals
## D = 0.069901, p-value = 0.0001514
## alternative hypothesis: two-sided
##
##
##  Box-Ljung test
##
## data:  interarrivals
## X-squared = 1.5802, df = 1, p-value = 0.2087
```

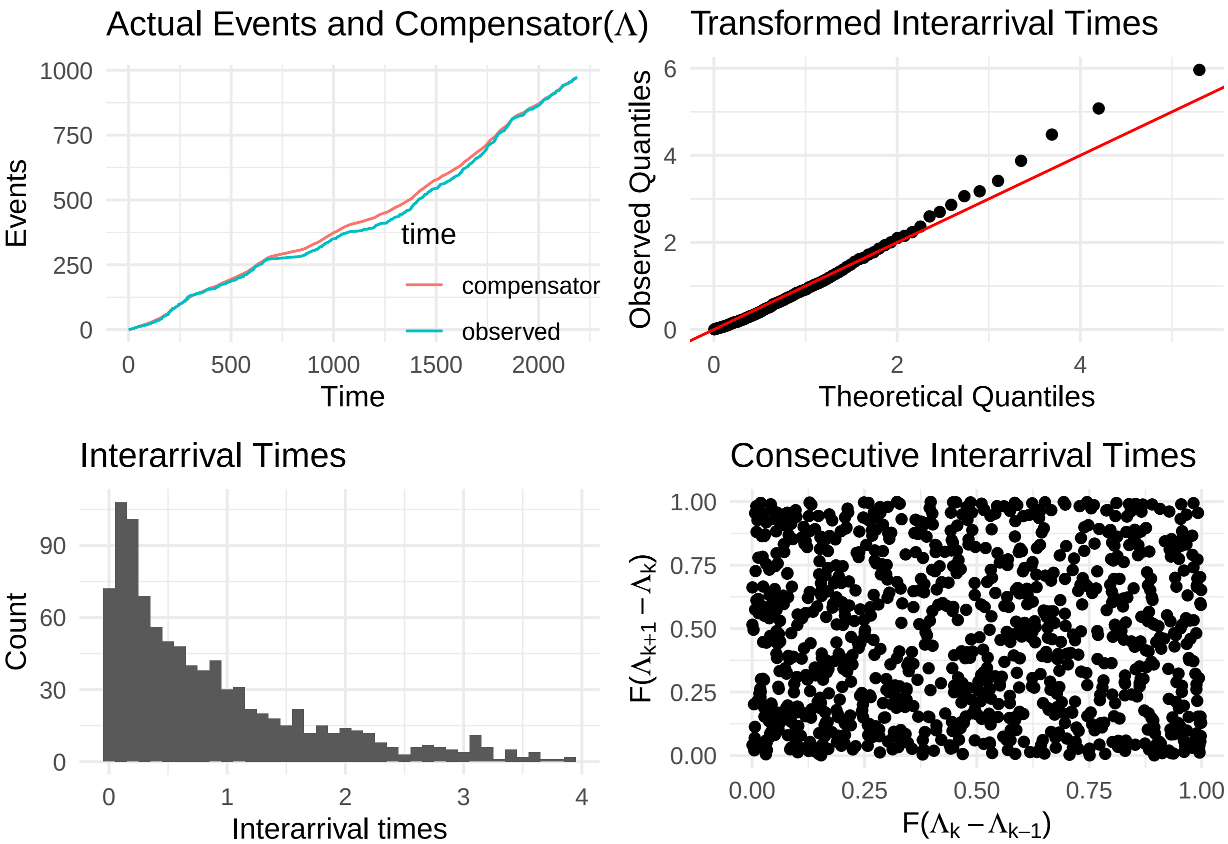


```r
par <- get_coefs(fit)[,1]
hawkes <- data.frame(date = times,
           int = stelfi:::hawkes_intensity(times = times, mu = par[1],
                                                    alpha = par[2], beta = par[3])) |>
  ggplot(., aes(x = date, y = int)) + geom_line() + geom_rug(sides = "b") +
    theme_classic() + ylab(expression(lambda(t))) + ylim(c(0,1.2)) +
  xlab("") + scale_x_continuous(breaks = c(0, 792 , 1460, 2188),
                                              labels = c(2000, 2002, 2004, 2006)) +
    theme(axis.text = element_text(size = 12),
              axis.title.y = element_text(size = 15))
hawkes
```

Using emhawkes

```r
## hspec class, same starting values as above
h <- new("hspec", mu = 0.1, alpha = 0.03, beta = 0.1)
## uses interarrival times
inter <- diff(times)
fit_em <- hfit(object = h, inter_arrival = inter)
## estimated parameter values & log-likelihood
## shown in Table 3
summary(fit_em)
```

```
## --------------------------------------------
## Maximum Likelihood estimation
## BFGS maximization, 86 iterations
## Return code 0: successful convergence
```

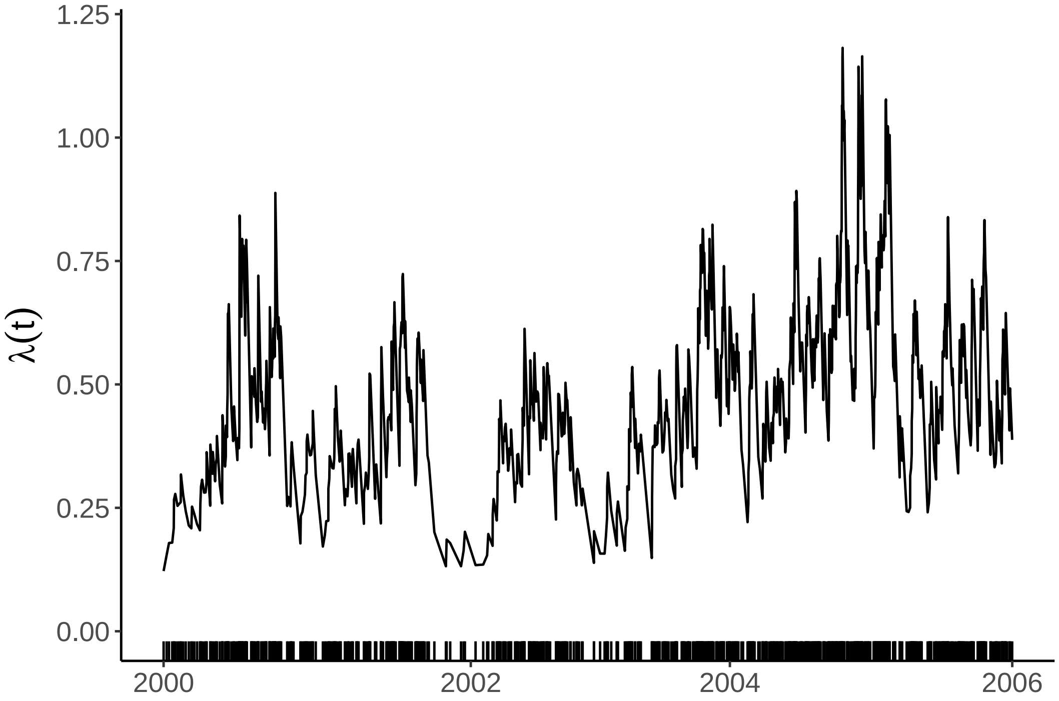

Figure 3: Figure in manuscript: Fitted Hawkes process of the reported temporal occurrence of Sasquatch sightings across the contiguous USA.

```
## Log-Likelihood: -1676.068
## 3  free parameters
## Estimates:
##         Estimate Std. error t value Pr(> t)
## mu1      0.11683    0.04982   2.345  0.0190 *
## alpha1   0.06692    0.03465   1.931  0.0534 .
## beta1    0.09082    0.05869   1.547  0.1218
## ---
## Signif. codes:  0 '***' 0.001 '**' 0.01 '*' 0.05 '.' 0.1 ' ' 1
## --------------------------------------------
```

Using `hawkesbow`

```
fit_bow <- mle(events = times, kern = "Exponential", end = max(times))
## note that in this formulation the second parameter is equivalent to
## alpha*beta above
## estimated parameter values
## shown in Table 3
fit_bow$par
```

```
## [1] 0.12172007 0.72979610 0.09071124
```

```
## Standard errors based on Hessian matrix
-fit_bow$model$ddloglik(times, max(times)) |>
    solve() |> diag() |> sqrt()
```

```
## [1] 0.04366237 0.10288730 0.05285163
```

```r
## log-likelihood (Table 2)
fit_bow$model$loglik(times, max(times))
```

```
## [1] -1681.156
```

Using hawkes

```r
par <- get_coefs(fit)[,1]
## negative log-likelihood
likelihoodHawkes(lambda0 = par[1], alpha = par[2], beta = par[3], history = times)
```

```
## [1] 1681.156
```

Fitting a spatial log-Gaussian Cox process

Using stelfi

```
## get sf of the contiguous US
sf_use_s2(FALSE)
us <- maps::map("usa", fill = TRUE, plot = FALSE) %>%
    sf::st_as_sf() %>%
    sf::st_make_valid()
## dataframe of sighting locations (lat, long)
locs <- sf::st_coordinates(sasquatch) %>%
  as.data.frame() %>%
  rename(., c("x" = "X", "y" = "Y"))
## Delauney triangulation of domain
smesh <- INLA::inla.mesh.2d(loc = locs[, 1:2], max.edge = 2, cutoff = 1)
## fit model with user-chosen parameter starting values
system.time(fit <- fit_lgcp(locs = locs, sf = us, smesh = smesh,
                                parameters = c(beta = 0, log_tau = log(1),
                                               log_kappa = log(1))))
```

```
##    user  system elapsed
##  31.029   0.794  31.942
```

```
## estimated parameter values
## shown in Table
get_coefs(fit)
```

```
##             Estimate Std. Error
## beta      -0.7659374  0.3558810
## log_tau   -0.6109840  0.1028264
## log_kappa -0.9540269  0.1510160
## range      7.3430015  1.1089108
## stdev      1.3491825  0.1480772
```

```
## expected num events
## get weights used, plot = TRUE in function will plot
w <- get_weights(smesh, sf = us)$weights
## extract field values at mesh nodes
f <- get_fields(fit, smesh)
sum(w * exp(f + get_coefs(fit)[1, 1])) ## true 972
```

```
## [1] 970.2504
```

Functions to plot fitted model and random field.

```
## plotting mesh weights
get_weights(smesh, sf = us, plot = TRUE)
```

```
## plot estimated intensity
show_lambda(fit, smesh, sf = us, clip = TRUE)
```

```
## random field (value at each mesh node)
f <- get_fields(fit, smesh, plot = TRUE)
```

```
## alternatively, pipe get_fields to show_field (note clip = TRUE)
get_fields(fit, smesh) |>
    show_field(smesh = smesh, sf = us, clip = TRUE)
```

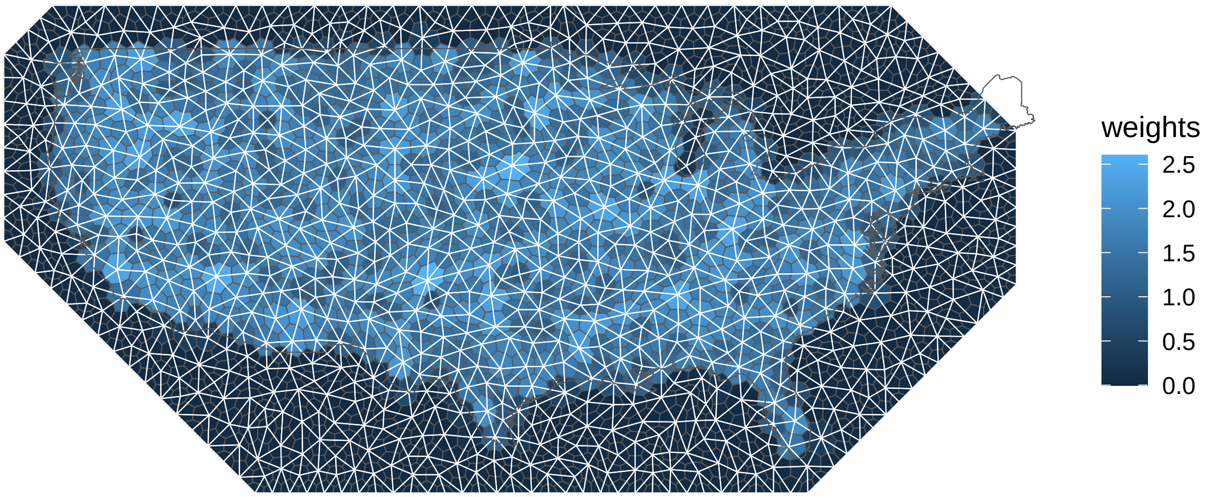

Figure 4: Delaunay triangulation (white) and resulting Voronoi tessellation (grey) where each Voronoi cell is coloured according to the integration weight used in the LGCP model.

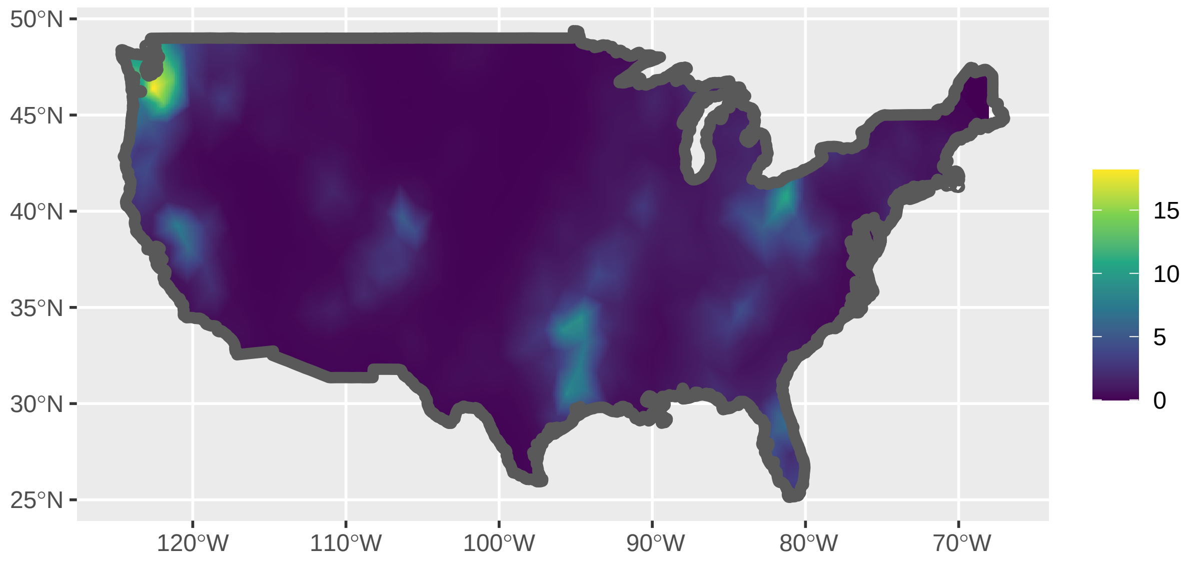

Figure 5: Estimated LGCP intensity from the fitted model for the reported Sasquatch sighting locations.

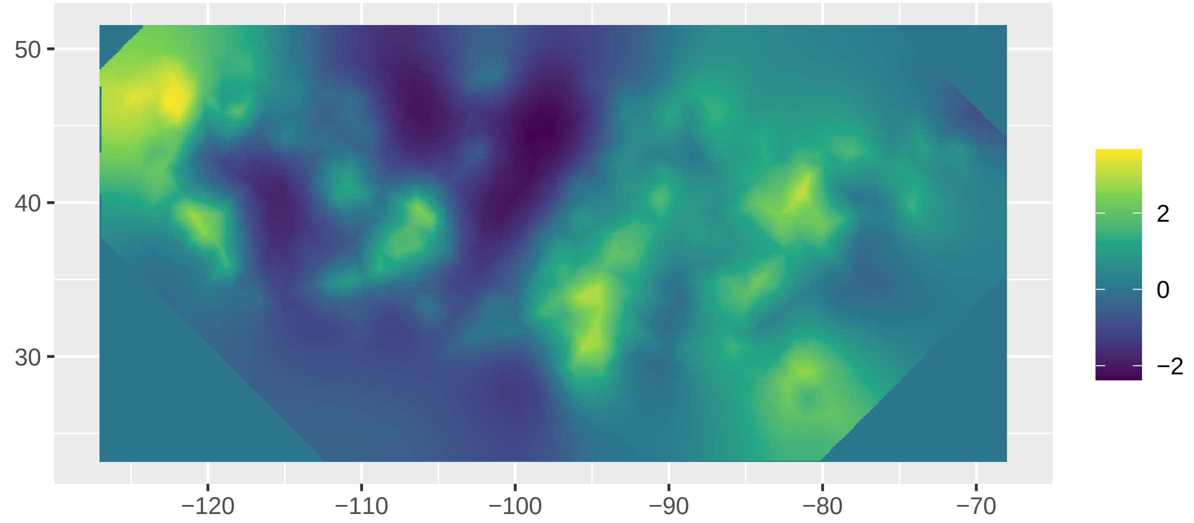

Figure 6: Estimated GMRF values from the fitted model for the reported Sasquatch sighting locations.

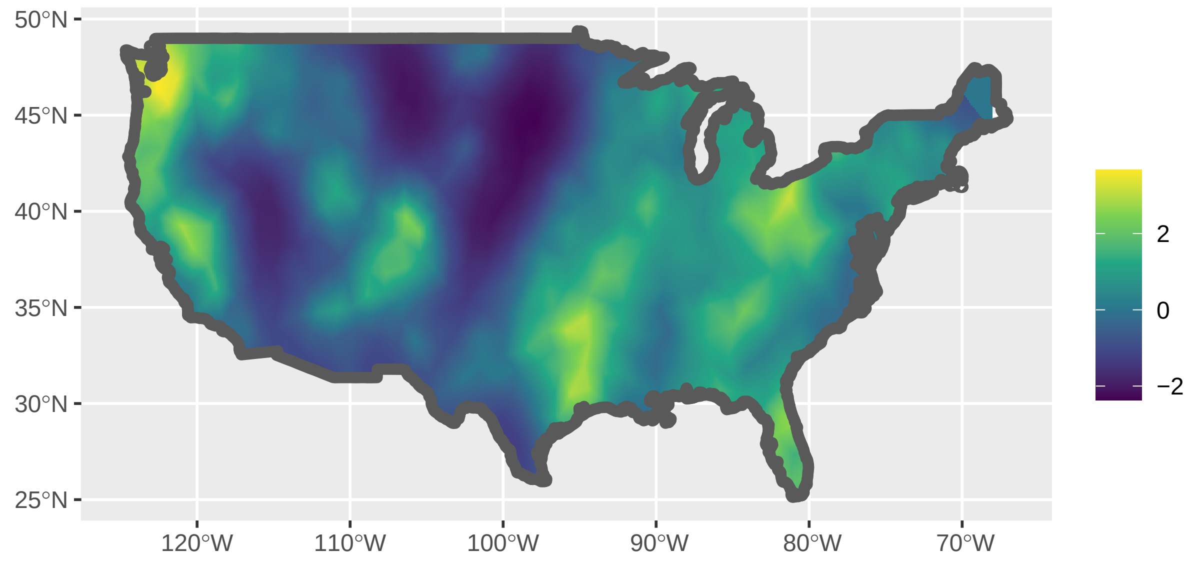

Figure 7: Estimated GMRF values from the fitted model for the reported Sasquatch sighting locations.

```r
## stelfi plots for manuscript
st <- show_lambda(fit, smesh, sf = us, clip = TRUE) +
        ggplot2::labs(fill = "") +
        ggplot2::xlab("") + ggplot2::ylab("") +
        ggplot2::scale_fill_viridis_c("Expected number \nof sightings", option = "D",
                                                                    limits = c(0,15)) +
        theme_void() + ggtitle("stelfi")
st
```

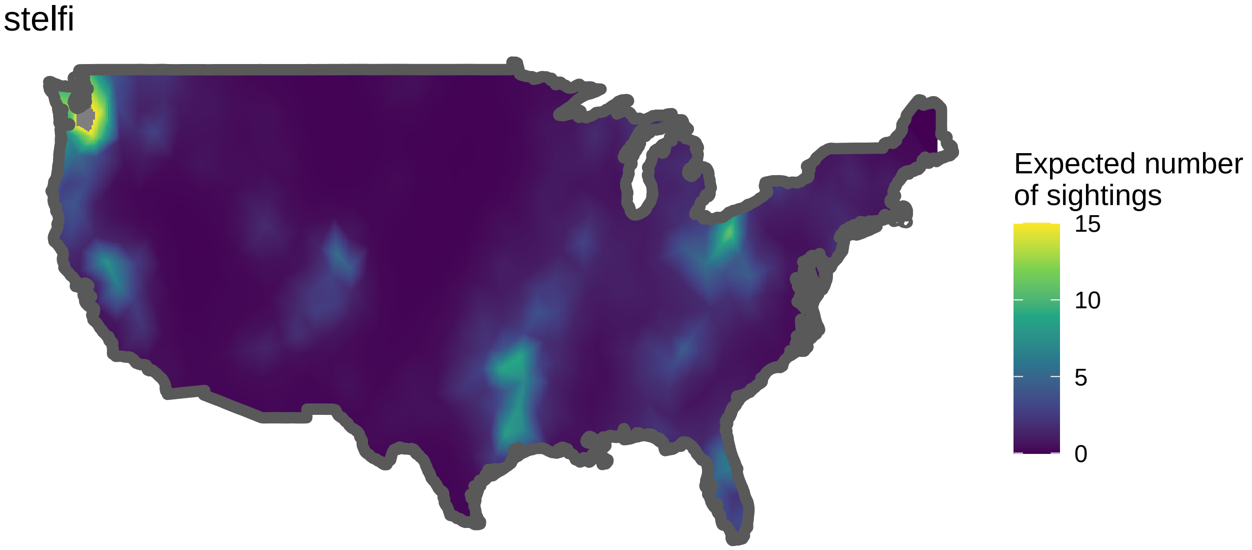


```r
wt <- get_weights(smesh, sf = us, plot = TRUE)
```

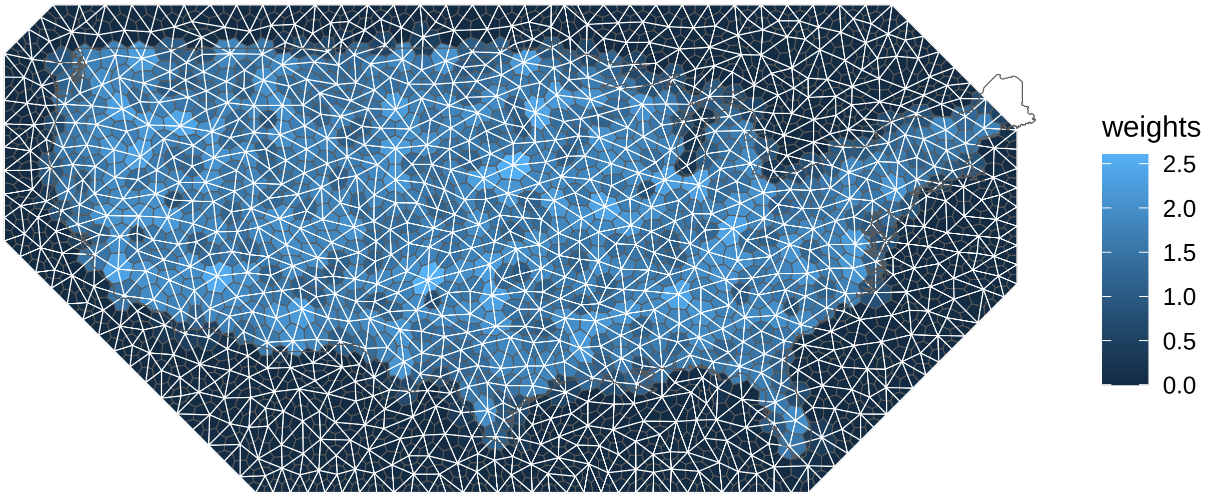


```r
wt
```

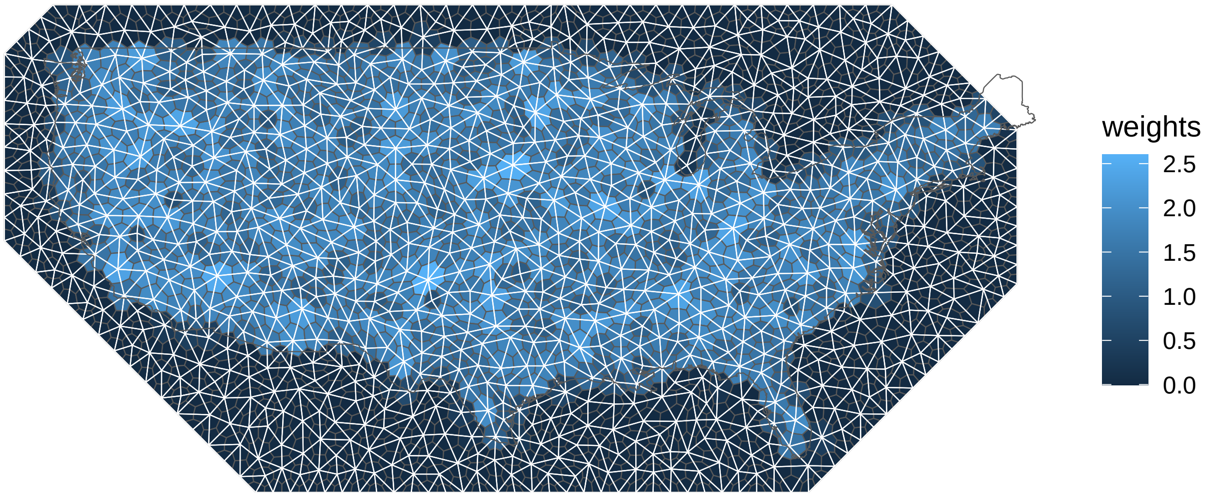

Using inlabru

```
set.seed(1234)
## following https://inlabru-org.github.io/inlabru/articles/web/2d_lgcp.html
## locations and domain as sp
locs_sp <- locs; coordinates(locs_sp) <- c("x", "y")
domain <- as(us, "Spatial")
matern <- inla.spde2.pcmatern(smesh,
  prior.sigma = c(0.1, 0.01),
  prior.range = c(5, 0.01)
)
## latent field
cmp <- coordinates ~ mySmooth(coordinates, model = matern) + Intercept(1)
proj4string(locs_sp) <- smesh$crs <- proj4string(domain)
## fit model
system.time(fit_inla <- lgcp(cmp, locs_sp, samplers = domain,
                                                      domain = list(coordinates = smesh)))
```

```
##    user  system elapsed
##  24.972   4.914  12.640
```

```
## estimated parameter values
## shown in Table 4
fit_inla$summary.fixed
```

```
##                 mean        sd 0.025quant   0.5quant   0.975quant       mode
## Intercept -0.4907638 0.2499805 -0.9902233 -0.4885046 -0.003929051 -0.4840847
##                    kld
## Intercept 3.900221e-09
```

```
fit_inla$summary.hyperpar
```

```
##                          mean         sd 0.025quant  0.5quant 0.975quant
## Range for mySmooth 7.9401368 0.81253926   6.491959 7.8863679   9.690657
## Stdev for mySmooth 0.8759888 0.06323398   0.759306 0.8731916   1.008437
##                          mode
## Range for mySmooth 7.7605645
## Stdev for mySmooth 0.8667776
```

```r
## overall prediction
Lambda <- predict(
  fit_inla,
  ipoints(domain, smesh),
  ~ sum(weight * exp(mySmooth + Intercept))
)
Lambda$mean ## true 972
```

```
## [1] 9826260
```

```r
## inlabru plot for manuscript
pred <- predict(
  fit_inla,
  pixels(smesh, mask = domain),
  ~ data.frame(
    lambda = exp(mySmooth + Intercept),
    loglambda = mySmooth + Intercept
  )
)
bru <- ggplot() +
  gg(pred$lambda) +
  gg(domain) +
  theme_void() +
  geom_sf(data = us, inherit.aes = FALSE, fill = NA) + coord_sf() +
        ggplot2::scale_fill_viridis_c("Expected number \nof sightings",
                                                                   option = "D", limits = c(0,15)) +
    ggtitle("inlabru")
bru
```

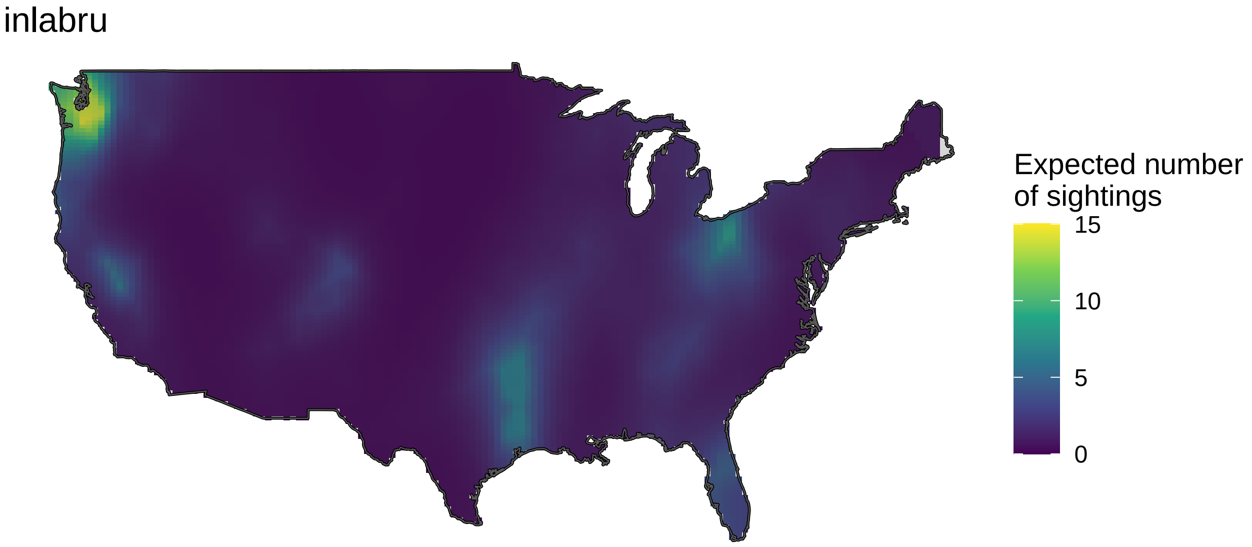

Marked model

In summary, Sasquatch sightings are categorised by the BFRO according to these criteria:

- Class A, clear sightings,
- Class B, observed at a great distance or in poor lighting conditions,
- Class C, second- and third-hand reports, or stories with an untraceable sources.

```
us <- maps::map("state", fill = TRUE, plot = FALSE) %>%
    sf::st_as_sf() %>%
    sf::st_make_valid()
## needed for GDAL shipped with older Ubuntu dist
sf::st_crs(sasquatch) <-  sf::st_crs(us) <-
    "+proj=longlat +datum=WGS84 +no_defs +ellps=WGS84 +towgs84=0,0,0"
## We ignore the one Class C report for the marked model
sasquatch %>%
  filter(classification != "Class C") |>
  ggplot() +
  geom_sf(aes(col = classification), alpha = 0.3) +
  coord_sf() +
  geom_sf(data = us, fill = NA) +
  theme_classic()
```

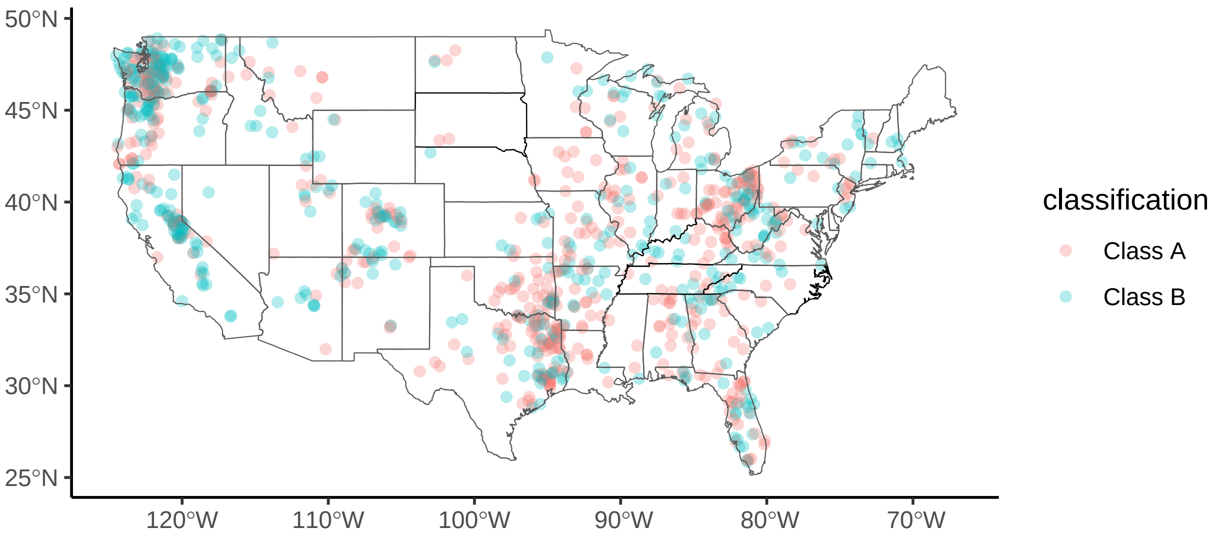


```
## removing proj4string (spatial package issues)
smesh$crs <- NULL
## modelling classification as a mark
idx <- which(sasquatch$classification != "Class C")
sas <- sasquatch[idx, ]
## Bernoulli, success = Class A
marks <- cbind(classification = ifelse(sas$classification == "Class A", 1 , 0))
## finding nearest covariate values at mesh node
c <- st_as_sf(x = data.frame(x = smesh$loc[,1], y = smesh$loc[,2]),
              coords = c("x", "y"), crs = st_crs(sas)) %>% elevatr::get_elev_point(., src = "aws")
covariates <- cbind(elevation = c$elevation/1000)
locs <- sf::st_coordinates(sas) %>%
  as.data.frame() %>%
  rename(., c("x" = "X", "y" = "Y")) %>%
    as.matrix()
```

```
parameters <- list(betamarks = matrix(0, nrow = 2, ncol = ncol(marks)),
                   log_tau = c(log(1), log(1)), log_kappa = c(log(1), log(1)),
                   marks_coefs_pp = rep(0, ncol(marks)), betapp = c(0, 0))
## a mark specific field and shared covariate & field
fit <- fit_mlgcp(locs = locs, marks = marks,
                 sf = us, smesh = smesh,
                 parameters = parameters, methods = 2, pp_covariates = 1,
                 marks_covariates = 1,
                 fields = 1, covariates = covariates)
## estimated parameter values
pars <- get_coefs(fit)
pars
```

```
##                   Estimate Std. Error
## betamarks       0.22743915 0.28432959
## betamarks      -0.37193922 0.17766579
## betapp         -0.82304146 0.35817863
## betapp          0.11358944 0.25117415
## marks_coefs_pp  0.04351647 0.06288582
## tau             0.20352848 0.02009822
## tau             2.34314993 1.94008889
## kappa           0.71471818 0.08818959
## kappa           0.23930772 0.15004934
```

Plot estimated shared random field.

```
sf_use_s2(FALSE)
f <- get_fields(fit, smesh)
## lgcp shared field
show_field(f[1:smesh$n], smesh = smesh, sf = us, clip = TRUE)
```

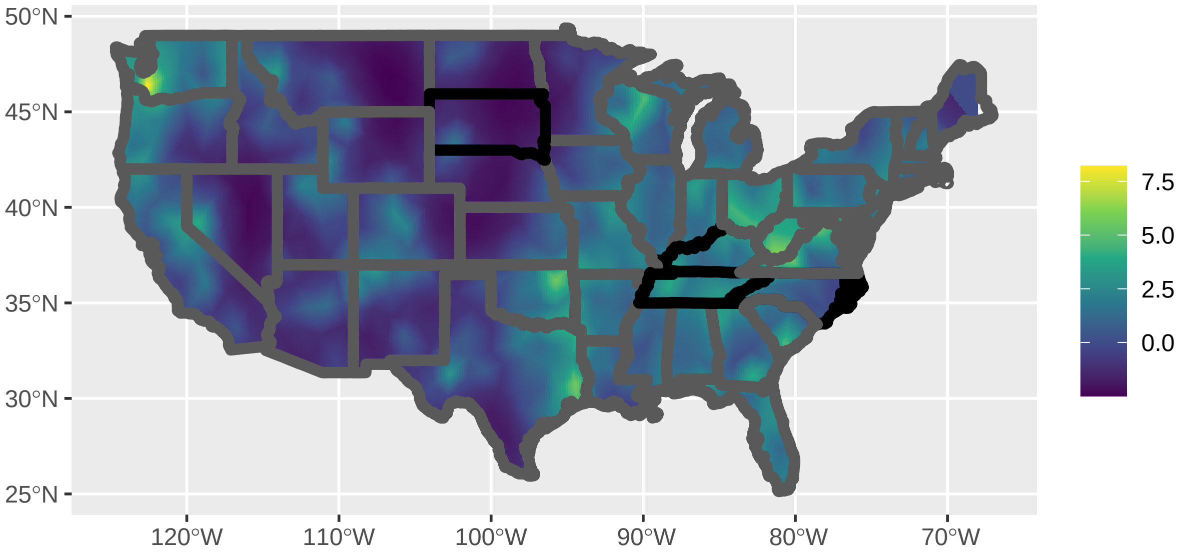


```
## mark specific field
sf_use_s2(FALSE)
show_field(f[(smesh$n+1):length(f)], smesh = smesh, sf = us, clip = TRUE)
```

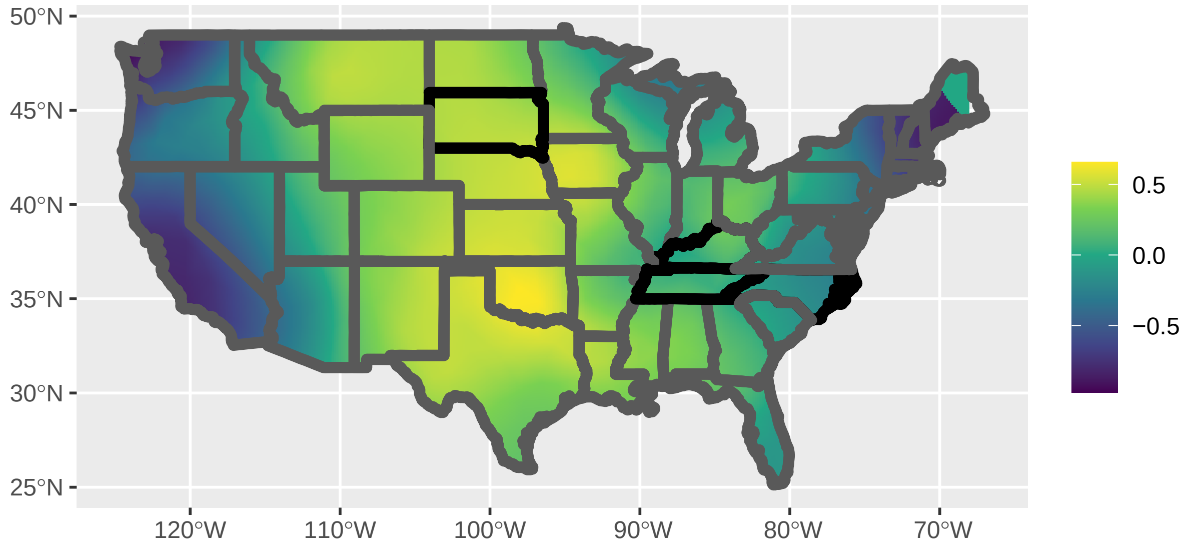

Spatiotemporal self-excitement

```
## data prep
## times must be unique and in ascending order
locs <- sf::st_coordinates(sasquatch) %>%
  as.data.frame() %>%
  mutate(t = difftime(sasquatch$date, min(sasquatch$date), units = "weeks")) %>%
    mutate(t = as.numeric(t)) %>%
  rename(., c("x" = "X", "y" = "Y"))
locs <- locs[!duplicated(locs[, c('t')]), ]
locs <- locs[order(locs$t), ]
loc <- data.frame(x = locs$x, y = locs$y)
times <- locs$t
sf_use_s2(FALSE)
us <- maps::map("usa", fill = TRUE, plot = FALSE) %>%
    sf::st_as_sf() %>%
    sf::st_make_valid()
## Delauney triangulation of domain
smesh <- INLA::inla.mesh.2d(loc = locs[, 1:2], max.edge = 2, cutoff = 1)
st_crs(us) <- NA
## spatiotemporal Hawkes process
param <- list(mu = 1, alpha = 3, beta = 6, xsigma = 0.2,
             ysigma = 0.2, rho = 0)

fit <- fit_stelfi(times = times, locs = loc, sf = us, smesh = smesh, parameters = param)
## Extract and print estimated parameters
pars <- get_coefs(fit)
## estimated parameter values & log-likelihood
## shown in Table
pars
```

```
##               Estimate   Std. Error
## mu      0.0006057541 6.809677e-05
## coefs  -7.4090364408 1.124165e-01
## alpha   0.0215078903 2.769752e-03
## beta    0.0215078907 2.769752e-03
```

```
## xsigma  0.7059711050 5.175609e-02
## ysigma  0.9131787587 9.134962e-02
## rho     0.1998886812 1.225789e-01
```
